# Immunosenescence remodels the osteoimmune microenvironment in osteoarthritis: from cellular crosstalk to regenerative therapeutics

**DOI:** 10.3389/fimmu.2026.1976649

**Published:** 2026-10-30

**Authors:** Ruofan Liu, Ziheng Zhu, Yunfei Li, Xiaoman Xing, Xinyang Wang, Chuanbing Huang

**Affiliations:** 1 Department of Rheumatology and Immunology, The First Affiliated Hospital of Anhui University of Traditional Chinese Medicine, Hefei, China; 2 School of Chinese Medicine, Bozhou University, Bozhou, China; 3 Key Laboratory of Xin’an Medicine, Ministry of Education, Anhui University of Chinese Medicine, Hefei, China

**Keywords:** cellular crosstalk, immunosenescence, microenvironmental remodeling, osteoarthritis, osteoimmunology, resolution of inflammation, senotherapeutics

## Abstract

Osteoarthritis (OA) has evolved from a condition conventionally regarded as mere mechanical “wear and tear” to a complex, whole-joint disorder driven by chronic low-grade inflammation and cellular dysfunction. Within the joint, the osteoimmune microenvironment—composed of articular cartilage, synovium, subchondral bone, the infrapatellar fat pad (IPFP), and resident stem cell niches—serves as the central site of osteoimmune interaction for maintaining tissue homeostasis and repair. Accumulating evidence indicates that age-associated dysregulation and senescence of innate and adaptive immune cell subsets represent major contributors to OA progression. Age- and senescence-associated immune states generate persistent inflammatory, oxidative, and damage-associated signals that contribute to microenvironmental deterioration. In this review, we propose a unified framework elucidating how immunosenescence reshapes the osteoimmune microenvironment via aberrant intercellular communication. We systematically delineate the distinct cellular crosstalk networks connecting immunosenescent drivers with chondrocytes, synovial fibroblasts, subchondral bone remodeling units, IPFP adipocytes, and mesenchymal stem/stromal cells (MSCs). Furthermore, we highlight emerging therapeutic avenues to reprogram this senescent niche, encompassing senotherapeutics, immunometabolic reprogramming, biomaterial-assisted targeted delivery, and engineered cell therapies. Finally, we propose the “Resolution Failure Hypothesis” as a testable mechanistic framework in which impaired efferocytosis and dysregulated specialized pro-resolving mediator (SPM) signaling may contribute to the persistence of osteoimmune remodeling. Rather than proposing defective inflammation resolution as a new biological phenomenon, this framework integrates established resolution-related mechanisms with age-associated immune remodeling and asks whether insufficient restoration of clearance and resolution capacity helps explain the incomplete structural recovery observed after senescent-cell-targeted interventions. Reconceptualizing OA through the lens of immunosenescent crosstalk will accelerate the translation of disease-modifying therapies from microenvironmental modulation toward functional joint regeneration.

## 1 Introduction

### 1.1 Paradigm shift: from mechanical degeneration to osteoimmune

Osteoarthritis (OA) remains a leading cause of chronic disability and persistent pain in the global aging population, posing a substantial socioeconomic burden (1), yet approved disease-modifying osteoarthritis drugs (DMOADs) that arrest its structural progression remain unavailable. Although historically characterized as an inevitable process of localized mechanical wear and tear, the pathophysiological paradigm of OA has undergone a profound conceptual shift. OA is now recognized as a heterogeneous, whole-joint disorder that integrates the entire articular apparatus into a biomechanically and immunologically coupled organ system (2).

Central to this paradigm is the osteoimmune microenvironment—a specialized anatomical and functional niche wherein skeletal components dynamically interact with resident and infiltrating immune cell networks (3). During physiological aging, this niche undergoes progressive age-associated immune remodeling, characterized by functional decline, phenotypic shifts, and immunosenescence across various immune cell compartments. While systemic low-grade inflammation (inflammaging) provides a permissive predisposing backdrop, age-associated immune remodeling may contribute substantially to remodeling intra-articular cellular composition and signaling cascades (4, 5).

Senescence-associated and age-remodeled immune states—predominantly senescent synovial macrophages (6), neutrophils displaying heightened NETosis (7), and senescence-associated CD4^+^CD28^-^ T cells (8) —continuously secrete a pathogenic repertoire of SASP components, reactive oxygen species (ROS), and matrix-degrading enzymes. Notably, joint destruction may not stem solely from the cell-autonomous accumulation of senescent cells, but rather from how immunosenescence reshapes intercellular interactions within the osteoimmune microenvironment. Here, we propose that immunosenescence may contribute to the remodeling, translating local immune stress into catabolic signaling cascades across articular cartilage, synovium, subchondral bone, the infrapatellar fat pad (IPFP) (9), and mesenchymal progenitor niches. Deciphering these complex intercellular communication networks holds promise for establishing precision regenerative paradigms aimed at therapeutically reprogramming the senescent osteoimmune microenvironment (10).

Existing literature has extensively characterized individual facets of joint aging, cataloging chondrocyte and synoviocyte cellular senescence, systemic inflammaging, immune-cell shifts, and localized osteoimmune interactions (3, 11–13). However, these biological processes are frequently evaluated as parallel, independent phenomena rather than components of a continuous, causally linked pathological cascade. Many current senescence-centered paradigms emphasize the progressive accumulation of senescent niche cells and the continuous emission of their pro-inflammatory senescence-associated secretory phenotype (SASP). Under this assumption, conventional translational strategies have focused heavily on eliminating senescent cells (senolysis) or neutralizing downstream inflammatory cytokines.

Yet, accumulating preclinical and clinical evidence reveals a pronounced therapeutic disconnect: although senolytics and cytokine-targeted approaches can attenuate nociceptive or inflammatory phenotypes in selected models, their ability to achieve consistent structural modification and durable restoration of joint homeostasis remains limited and heterogeneous (14) (15–18). This therapeutic stalemate exposes a fundamental, unaddressed knowledge gap in the field: Under what conditions might senescent-cell clearance fail to produce durable structural repair, and could insufficient restoration of inflammation-resolution capacity contribute to this limitation?

In this review, we move beyond descriptive multidisciplinary integration to propose a unified framework that orders these processes into a testable sequence. Rather than framing immunosenescence merely as the accumulation of aged leukocytes, we conceptualize it as age-associated remodeling of intercellular communication across articular cartilage, synovium, subchondral bone, the IPFP, and mesenchymal progenitor niches. We propose that inadequate endogenous inflammation resolution may represent a mechanistic bridge between altered immune-cell states, pathological intercellular crosstalk, and persistent whole-joint remodeling. Candidate contributors include impaired macrophage efferocytic capacity involving MerTK/Axl and dysregulated specialized pro-resolving mediator (SPM) signaling; however, whether these abnormalities causally sustain OA progression or explain limited structural recovery following senolysis remains to be established.

Within this framework, resolution deficiency is not presented as a proven primary cause of OA, but as a potentially important amplifier of persistent tissue injury and a testable barrier to restoration of homeostasis. The proposed sequence—from age-associated immune-state remodeling through dysregulated intercellular communication and insufficient inflammatory termination to ongoing structural damage—motivates experimental evaluation of approaches combining senescent-cell targeting with restoration of resolution capacity, rather than presupposing their disease-modifying efficacy.

**Figure 1** provides the conceptual framework underlying this review. Rather than positioning immunosenescence as an isolated cause of OA, we propose that age-associated cellular stress, metabolic dysfunction, chronic low-grade inflammation, repeated tissue injury, and mechanical stress converge to remodel immune-cell states within the osteoimmune microenvironment. These altered cellular states generate pathological signals and rewire intercellular communication across cartilage, synovium, subchondral bone, the IPFP, and mesenchymal progenitor niches, ultimately contributing to whole-joint OA remodeling.

**Figure 1:**
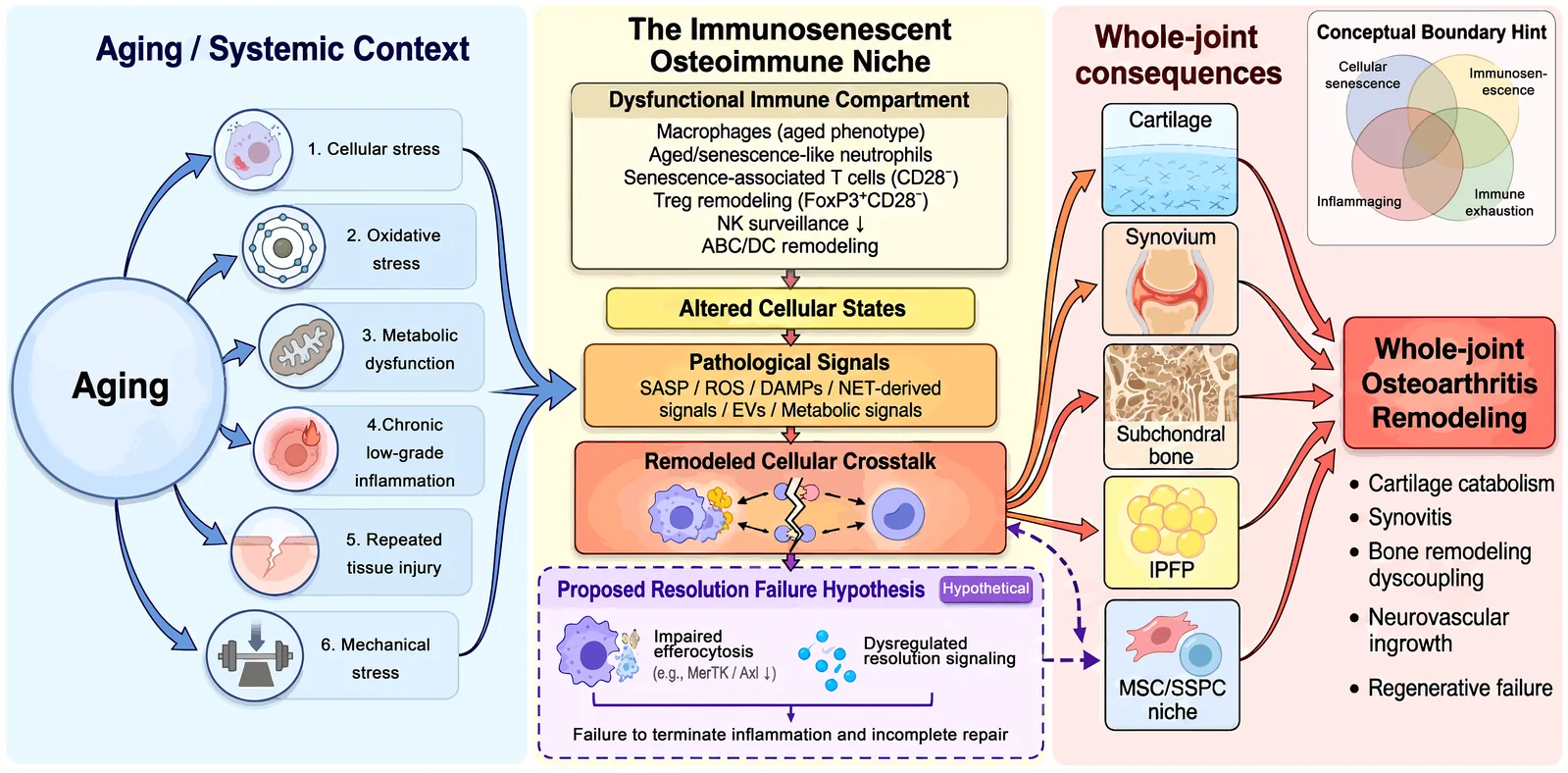
Conceptual framework linking aging, immunosenescence, impaired inflammation resolution, and osteoimmune remodeling in osteoarthritis.

This framework also distinguishes immunosenescence from cellular senescence, inflammaging, and immune exhaustion, which may overlap but represent biologically distinct processes.

### 1.2 Conceptual boundaries: distinguishing cellular senescence, immunosenescence, inflammaging, and exhaustion

To establish a rigorous mechanistic foundation for understanding joint aging, it is essential to delineate the conceptual boundaries separating cellular senescence, immunosenescence, inflammaging, and immune exhaustion within the osteoimmune microenvironment. While these biological processes frequently intersect during OA progression, they represent distinct phenomena arising through different mechanisms and should not be used interchangeably (3, 19).

#### 1.2.1 Cellular senescence

Refers to a cell-autonomous stress-response state characterized by durable cell-cycle arrest, altered metabolic activity, apoptosis resistance, and a context-dependent SASP (20, 21). Importantly, no single marker (such as p16INK4a, p21CIP1, or SA-β-gal) uniquely defines cellular senescence in isolation (22, 23). In skeletal and immune tissues, defining cellular senescence requires a composite panel incorporating cell identity, DNA-damage markers (e.g., γH2AX), SASP profiling, and functional proliferative loss.

#### 1.2.2 Immunosenescence

Denotes a broader, tissue- and system-level age-associated remodeling of the immune compartment (11). It encompasses structural shifts such as naïve lymphocyte pool contraction, terminally differentiated memory cell expansion, myeloid skewing, altered immune surveillance, and attenuated efferocytosis or resolution responses. Immunosenescence describes the altered functional state of the immune system as a whole, rather than requiring that every constituent immune cell undergo classic cell-autonomous cell-cycle arrest (12).

#### 1.2.3 Inflammaging

Describes a chronic, low-grade, age-associated inflammatory microenvironmental state (5, 24). Driven by convergent inputs, including SASP emission, metabolic stress, damage-associated molecular patterns (DAMPs), mechanical overload, and dysbiosis. Inflammaging characterizes the inflammatory tone of the tissue milieu. Elevated cytokine readouts (e.g., IL-6, TNF-α, CRP) reflect an inflammaging background but do not independently prove cell-intrinsic senescence.

#### 1.2.4 Immune exhaustion

Represents a distinct functional state arising predominantly from persistent, chronic antigen stimulation. Exhaustion is defined by the elevated expression of inhibitory checkpoint receptors (e.g., PD-1, TIM-3, LAG-3) accompanied by a progressive, hierarchical loss of effector cytokine capacity (25). In contrast, immunosenescence-associated T-cell phenotypes (e.g., CD4+CD28- T cells) often retain or hyper-activate their capacity to secrete pro-inflammatory SASP factors despite costimulatory receptor loss (26).

Clarifying these conceptual boundaries ensures that age-related osteoimmune dynamics are evaluated with mechanistic precision, preventing the oversimplification of complex immune phenotypes during joint degeneration (**Table 1**).

**Table 1:** Conceptual distinctions among cellular senescence, immunosenescence, inflammaging, and immune exhaustion.

Concept	Biological level	Core feature	Typical indicators	Relationship to OA	Ref.
Cellular senescence	Individual cell	Stable growth arrest + altered function/SASP	p16, p21, SA-β-gal, γH2AX, SASP	Chondrocyte/Synovial/immune-cell dysfunction	(20–23)
Immunosenescence	Immune system	Age-related immune remodeling	naïve T-cell loss, memory expansion, myeloid skewing, impaired surveillance	Altered immune-cell crosstalk	(11, 12)
Inflammaging	Tissue/systemic milieu	Chronic low-grade inflammation	IL-6, TNF-α, CRP, etc.	Persistent inflammatory microenvironment	(5, 24)
Immune exhaustion	Immune-cell functional state	Chronic antigen-induced dysfunction	PD-1, TIM-3, LAG-3, impaired effector function	May coexist with chronic inflammatory stimulation	(25, 26)

## 2 Cellular basis of immunosenescence in the osteoimmune microenvironment

Cellular senescence within the immune system is increasingly understood not merely as a loss of cellular viability, but as a profound phenotypic, metabolic, and functional remodeling. Within the osteoimmune microenvironment, age-related metabolic stress, oxidative damage, and persistent inflammatory stimuli drive immune subsets toward senescence-associated or dysfunctional states. These states are heterogeneous and should not be assumed to represent classical cellular senescence in every immune population. Delineating these distinct cellular alterations is essential for understanding how age-related immune remodeling may influence intercellular communication networks within the joint. **Table 2** summarizes key immunosenescence-associated phenotypes and corresponding markers across immune subsets in OA.

**Table 2:** Immunosenescence-Associated phenotypes of immune cell subsets in the OA osteoimmune microenvironment.

Immune cell subset	Immunosenescence-associated phenotype^a^	Key functional alteration	Microenvironmental impact in OA	Evidence level in OA	Ref.
Synovial Macrophages	p16^INK4a^, p21^CIP1^, SA-β-gal, γH2AX; MerTK/Axl/TIM-4 ↓	Impaired efferocytosis; pro-inflammatory polarization bias; sustained SASP	Secondary necrosis, DAMP accumulation, chronic synovitis	Strong	(6, 27–34)
Aged/senescence-like neutrophils	CXCR4 ↑, CD62L ↓; PAD4-dependent hypercitrullination (Cit-H3)	Heightened NET release; context-dependent ROS remodeling; prolonged tissue retention	Cartilage matrix cleavage, chondrocyte stress, amplified local inflammation	Emerging	(35–44)
Senescence-associated T cells	CD4^+^CD28^-^, CD57^+^, KIR/KLRG-1^+^; telomere attrition, γH2AX	Cytotoxic/SASP-shifted effector program; retained pro-inflammatory cytokine output	RANKL-driven osteoclastogenesis, synovial and matrix degradation	Emerging	(45–50)
Regulatory T cells (Tregs)	CD4^+^FoxP3^+^CD28^-^ subset; γH2AX accumulation, low TREC	Weakened suppression; increased Th17-like plasticity	Reduced restraint of local inflammation	Emerging	(53–55)
Age-associated B cells (ABCs)	CD11c^+^, T-bet^+^, CD21^low,^CD23^-^	Autoantibody and pro-inflammatory cytokine production; altered RANKL/OPG balance	Humoral immune dysregulation, bone-resorptive bias	Limited	(59–62)
NK cells	CD56^bright^ ↓/CD56^dim^ CD57^+^ ↑ (human); NKG2D, NKp46 altered	Reduced senescent-cell surveillance; impaired degranulation and cytokine output	Escape and accumulation of senescent stromal cells	Limited	(56–58)
Dendritic cells	Altered CCR7 expression; dysregulated antigen presentation	Impaired CCL21-directed migration; pro-inflammatory cytokine skewing	Defective tolerance, sustained low-grade inflammaging	Limited	(63–65)

a Species note: The ABC phenotype (CD11c^+^ T-bet^+^ CD21^low^ CD23^-^) was primarily defined in murine models; human correlates remain under investigation. NK-cell markers listed are human-centric (CD56); murine equivalents include NK1.1 and NKp46. No single marker uniquely defines immunosenescence; phenotypes should be interpreted as composite panels.

The ‘Strong’ designation reflects direct human OA evidence for impaired macrophage efferocytosis together with complementary experimental evidence for senescence-associated macrophage phenotypes; direct human validation of the complete composite senescence phenotype remains limited. The symbol "↑" indicates an increase or upregulation, whereas "↓" indicates a decrease or downregulation in expression, abundance, or frequency relative to the relevant comparator.

### 2.1 Senescence-associated macrophage states

During aging and sustained microenvironmental stress, macrophages acquire classical hallmarks associated with cellular senescence, including elevated p16INK4a and p21CIP1 expression, enhanced senescence-associated β-galactosidase (SA-β-gal) activity, and persistent DNA damage foci (γH2AX); however, none of these markers alone is uniquely specific to macrophage senescence (27–29). Functional alterations in senescent macrophages primarily encompass three interrelated dimensions:

#### 2.1.1 Dysregulated inflammatory programs and loss of polarization plasticity

Healthy macrophages exhibit remarkable functional plasticity, dynamically transitioning between pro-inflammatory and tissue-repair phenotypes in response to microenvironmental cues. Although the conventional M1/M2 classification provides a useful operational framework, macrophage states in OA are highly heterogeneous and context-dependent. In senescent macrophages, this regulatory plasticity appears markedly impaired. Driven by basal NF-κB and p38 MAPK pathway activation, altered metabolic fluxes, and chromatin accessibility remodeling, senescent macrophages maintain a sustained pro-inflammatory profile (30). Concurrently, their capacity to adopt an anti-inflammatory or pro-resolving phenotype upon encountering resolution signals is attenuated (31).

#### 2.1.2 Impaired efferocytosis and clearance capacity

A critical functional defect observed in senescent macrophages is the decline in efferocytosis. Key apoptotic recognition receptors, including TIM-4 and the TAM receptor tyrosine kinase family members MERTK and AXL, frequently undergo dysregulation during aging and macrophage dysfunction (32). When efferocytic clearance is delayed, uncleared apoptotic cells accumulate within the microenvironment and can undergo secondary necrosis, thereby amplifying the local availability of endogenous DAMPs and inflammatory mediators (33).

#### 2.1.3 SASP secretion and low-grade inflammation

Senescent macrophages represent a major local source of SASP factors. Mediated in part through cytosolic DNA sensing and sustained NF-κB activation, these cells continuously produce pro-inflammatory cytokines (IL-1β, IL-6, TNF-α), chemokines (CCL2, CXCL8), and matrix metalloproteinases (MMP-1, MMP-9, MMP-13). Furthermore, mitochondrial dysfunction and elevated mitochondrial reactive oxygen species (mtROS) reinforce inflammatory cascades, sustaining SASP emission in senescent cells (6, 34).

### 2.2 Aged and senescence-like neutrophils

As the most abundant innate immune responders, neutrophils participate in the early phases of tissue injury and inflammatory responses. Rather than equating physiological neutrophil aging directly to classical cellular senescence, it is more accurately conceptualized as an age-associated state accompanied by continuous phenotypic, migratory, and functional remodeling. Previous studies have demonstrated that aged or senescence-like neutrophils exhibit altered surface receptor profiles, such as CXCR4 upregulation and remodeled CXCR2-dependent signaling, which influence their circulatory retention, tissue recruitment, and bone marrow homing kinetics (35, 36). Under chronic inflammation or tissue injury, this age-associated functional remodeling can foster neutrophil subsets endowed with enhanced pro-inflammatory and tissue-destructive potential.

#### 2.2.1 Heightened NETosis susceptibility

Neutrophil extracellular traps (NETs) are web-like structures composed of decondensed chromatin strands complexed with granule proteins. Evidence indicates that age-associated neutrophils exhibit an augmented propensity for NET formation under inflammatory or pathological stimulation, suggesting that aging may lower the activation threshold for NETosis—a process intimately linked to peptidylarginine deiminase 4 (PAD4)-mediated histone hypercitrullination (37, 38). Importantly, PAD4-dependent NETosis is not unique to aged neutrophils but represents a general mechanism governing NET formation across diverse inflammatory contexts. Thus, age-associated neutrophils, pre-existing NET-forming programs are more likely amplified within chronic inflammatory and pathological microenvironments. If NETs are not cleared in a timely manner, their associated components—including DNA, histones, myeloperoxidase (MPO), and neutrophil elastase (NE)—persist within the extracellular milieu, amplifying local inflammation and tissue destruction through pattern recognition receptors and downstream inflammatory pathways (39).

#### 2.2.2 Elevated ROS generation and oxidative stress

Age-associated neutrophils also undergo marked oxidative metabolic reprogramming. Notably, mitochondrial dysfunction, elevated mtROS production, and mitochondrial DNA (mtDNA) leakage are recognized as prominent features of the pathogenic phenotype of age-associated neutrophil subsets (39, 40). These alterations enhance oxidative stress, promote NET formation, and exert bystander effects on neighboring cells via the release of oxidatively modified molecules and mtDNA-associated danger signals (41). However, ROS production in aged neutrophils is not uniformly elevated; respiratory burst capacity is modulated by aging, inflammatory tone, and metabolic context (42). Therefore, rather than characterizing elevated ROS or NOX2 activation as a universal feature of all aged neutrophils, it is more appropriately understood as a context-dependent oxidative metabolic reprogramming shaped by aging, local inflammation, and metabolic cues (43). Moreover, altered survival signaling under chronic inflammatory conditions may prolong the persistence of aged or dysfunctional neutrophils in tissue niches, further sustaining chronic inflammation (44).

### 2.3 Senescence-associated T-cell states

T cells undergo extensive age- and stress-associated remodeling, representing a key adaptive immune feature of immunosenescence within the osteoimmune microenvironment. With advancing age and chronic antigenic stimulation, the naïve T-cell repertoire contracts while highly differentiated, antigen-experienced T-cell populations accumulate, accompanied by telomere shortening, DNA damage accumulation, metabolic reprogramming, and altered costimulatory receptor expression (45). Notably, T-cell immunosenescence is not fully synonymous with classical cellular senescence; for instance, although CD28 loss represents a hallmark of age-associated T-cell remodeling, CD4^+^CD28^-^ T cells can retain proliferative and effector capabilities under appropriate stimulation and are thus best conceptualized as senescence-associated or senescence-like T cells (46).

#### 2.3.1 Accumulation of CD4^+^CD28^-^ senescence-associated cytotoxic T cells

The progressive downregulation of CD28 is a classic phenotype of human T-cell immunosenescence. Under physiological conditions, CD28 engagement is required for robust T-cell activation and survival. Repeated antigenic stimulation and replicative history downregulate CD28 expression through transcriptional and epigenetic mechanisms, fostering the accumulation of highly differentiated CD4^+^CD28^-^ T cells (47). Rather than being functionally inert, these cells undergo substantial functional remodeling, acquiring phenotypes typical of cytotoxic lymphocytes, including the expression of NKG2D, CD57, and killer cell immunoglobulin-like receptors, alongside cytolytic machinery such as perforin and granzyme B (48, 49). In chronic inflammatory diseases, CD4^+^CD28^-^ T cells also demonstrate an enhanced capacity for pro-inflammatory cytokine production. Furthermore, CD4^+^CD28^-^ T cells can express high levels of RANKL to promote osteoclastogenesis, providing direct mechanistic evidence linking T-cell immunosenescence to bone resorption and osteoimmune imbalance (50).

#### 2.3.2 Distinguishing immunosenescence from T-cell exhaustion

Differentiating T-cell immunosenescence from T-cell exhaustion is essential for understanding chronic inflammatory microenvironments. T-cell exhaustion arises predominantly in chronic viral infections and tumor microenvironments subjected to persistent antigen exposure, characterized by sustained expression of inhibitory checkpoint receptors (e.g., PD-1, TIM-3, LAG-3) and a hierarchical loss of cytokine production, proliferation, and cytolytic activity driven by distinct transcriptional and epigenetic programs (25, 51). Conversely, senescence-associated T-cell states correlate more closely with extensive replicative history, telomere erosion, DNA damage, and metabolic reprogramming (45). Nevertheless, these two states can partially overlap phenotypically in chronic inflammatory niches; thus, they should not be distinguished solely by individual surface markers, but rather through integrated assessments of proliferative capacity, metabolic state, transcriptional circuitry, and functional competence (52).

#### 2.3.3 Treg functional remodeling and inflammatory plasticity

Regulatory T cells (Tregs) maintain self-tolerance, suppress excessive inflammation, and promote tissue repair, serving critical immunoregulatory functions within the osteoimmune niche. Chronic inflammation and age-associated immune remodeling can alter Treg phenotype and suppressive efficacy. In arthritic settings, studies have identified CD4^+^FoxP3^+^CD28^-^ Treg-like cells that harbor overlapping regulatory and senescence-associated features, including CD25, CTLA-4, PD-1, and FOXP3 expression, paired with low TREC levels and γH2AX accumulation, indicating that Tregs can also acquire senescence-associated characteristics (53). Furthermore, pro-inflammatory cytokine milieus influence Treg stability and function. Signals such as IL-6 and IL-1β can disrupt FOXP3-driven regulatory programs and promote functional remodeling toward a Th17-like inflammatory state (54). Consequently, sustained inflammation and oxidative stress can weaken Treg-mediated immunosuppression and increase Treg–Th17 plasticity, diminishing their ability to restrain local inflammatory responses. Notably, this Treg–Th17 plasticity should not be interpreted simply as irreversible lineage transdifferentiation, but rather as dynamic functional reprogramming between regulatory and effector programs in inflamed microenvironments (55).

### 2.4 Other immune populations

While macrophages, neutrophils, and T cells represent the most extensively characterized immune populations in the osteoimmune microenvironment, other resident and infiltrating immune subsets undergo age-associated phenotypic and functional remodeling, exacerbating local microenvironmental imbalance through altered immune surveillance, humoral immunity, and antigen presentation.

#### 2.4.1 Natural killer cells

Natural killer (NK) cells are innate lymphocytes vital for maintaining tissue immune surveillance and eliminating abnormal or senescent cells. With advancing age, NK cells undergo substantial subset and functional remodeling, typically manifested as a decline in the immunoregulatory CD56bright subset and a relative expansion of the highly differentiated CD56dim CD57^+^ population (56, 57). Although a proportion of CD56dim NK cells retain robust cytolytic activity, age-associated NK-cell remodeling is accompanied by altered activating receptor repertoires, diminished cytokine production, and impaired degranulation, with NKG2D, NKp46, and their downstream signaling pathways often compromised (58). This functional remodeling may weaken NK-cell-mediated surveillance and clearance of senescent or stressed cells, permitting senescent cell accumulation and sustaining low-grade chronic inflammation. Thus, NK-cell immunosenescence may contribute to the elevated senescent cell burden in the osteoimmune microenvironment by impairing senescent-cell surveillance.

#### 2.4.2 Age-associated B cells

A defining hallmark of B-cell immunosenescence is the progressive accumulation of age-associated B cells (ABCs). ABCs represent a distinct B-cell subset characterized by a unique transcriptional and phenotypic profile (typically CD11c^+^, CD11b^+^, T-bet^+^, CD21^-^, and CD23^-^), driven by convergent BCR, TLR7/TLR9, and IFN-γ signaling through a T-bet-dependent program. ABCs do not represent classical senescent cells in a cell-cycle-arrest sense, but rather an age-associated differentiation state that can also expand in chronic infections and autoimmune disorders (59, 60). In pathological inflammatory environments, ABCs display heightened antigen capture and presentation capacities, and produce autoantibodies and pro-inflammatory cytokines, thereby promoting chronic inflammation and the breakdown of immune tolerance (61). At the osteoimmune interface, B cells can also modulate osteoclastogenesis by altering the balance between RANKL and OPG production, suggesting that age-associated B-cell remodeling participates in bone resorption and skeletal dyshomeostasis (62).

#### 2.4.3 Dendritic cells

Dendritic cells (DCs) serve as key antigen-presenting cells bridging innate and adaptive immunity. Analogous to other immune subsets, age-associated alterations in DCs are highly subset- and tissue-dependent rather than reflecting a unidirectional functional decline. Studies show that aged DCs exhibit varying degrees of alterations in antigen uptake, migration, antigen presentation, and T-cell priming capacities (63) For instance, aged murine DCs exhibit impaired CCR7-dependent migration and blunted chemotactic responses toward CCL21, which may impair antigen transport to draining lymph nodes and dampen adaptive immune priming (64). Concurrently, aged human DCs can display increased secretion of pro-inflammatory cytokines, reduced output of immunoregulatory cytokines, and enhanced reactivity toward self-antigens, potentially contributing to chronic inflammation and compromised immune tolerance (65). Therefore, DC immunosenescence is more appropriately defined as a functional reprogramming of antigen presentation, migration, and immunoregulatory properties rather than a simple loss of function.

#### 2.4.4 Summary: formation of the immunosenescent microenvironment

The cell-autonomous dysfunctions detailed across these individual immune compartments do not occur in isolation; rather, they converge to establish a self-reinforcing immunosenescent microenvironment. In this compromised niche, pro-inflammatory macrophages, aged neutrophils with heightened NET-forming capacity, CD4^+^CD28^-^ senescence-associated T cells, functionally remodeled Tregs, and surveillance-impaired NK cells collectively sustain continuous inflammatory inputs. Concurrently, ABCs and remodeled DCs further amplify local immune imbalance through autoantibody production, altered antigen presentation, and aberrant immunoregulation.

The evidence base underpinning **Table 2** is deliberately graded: direct human OA data exist for synovial macrophages, whereas neutrophil and T-cell axes rely on arthritis-context association, and NK-, ABC-, and DC-related immunosenescence in OA remains largely extrapolated from aging biology. This heterogeneity should guide, rather than discourage, future subset-specific interrogation of the OA joint.

Together, these dysregulated immune subsets transform an otherwise regulated tissue niche into a chronic, self-sustaining inflammatory ecosystem. The persistent release of SASP factors, ROS, and DAMPs continuously impinges on neighboring structural cells, establishing the biological foundation for widespread perturbations in intercellular crosstalk across the entire osteoimmune microenvironment.

Importantly, the significance of these age-associated immune alterations lies not only in the intrinsic dysfunction of individual immune subsets, but also in the pathological signals they introduce into the joint microenvironment. Senescence-associated macrophage states can generate SASP factors, ROS, and defective efferocytic responses; neutrophil remodeling can increase NET-derived DNA, proteases, and oxidative stress; and senescence-associated or age-remodeled lymphocyte populations can alter cytokine production, immune regulation, and RANKL-dependent bone signaling. Together, these changes constitute a set of persistent inflammatory, metabolic, and clearance-related inputs that are transmitted to neighboring structural and stromal cells. Section 3 therefore examines how these altered immune states are translated into compartment-specific intercellular communication, tissue dysfunction, and self-reinforcing feedback across the whole joint.

## 3 Immunosenescence remodels cellular crosstalk in the osteoimmune microenvironment

OA progression reflects not only cell-intrinsic dysfunction within individual cell populations, but also the continuous remodeling of intercellular communication networks throughout the joint. A healthy joint relies on coordinated, bidirectional signaling among immune cells, synovial fibroblasts, chondrocytes, bone-lineage cells, and the extracellular matrix to maintain matrix homeostasis, tissue barrier integrity, and debris clearance. With advancing age and chronic inflammatory accumulation, shifts in immune cell composition, metabolic status, and secretory profiles reconfigure these paracrine and extracellular vesicle-mediated communication networks.

In the age-remodeled osteoimmune microenvironment, sustained production of pro-inflammatory cytokines, ROS, DAMPs, NET-derived components, and extracellular vesicles (EVs) acts on neighboring structural cells, shifting them from homeostatic maintenance toward inflammatory, stressed, and catabolic states. Reciprocally, damaged chondrocytes and other tissue cells release matrix degradation products and EVs, further stimulating immune cells and amplifying the inflammatory cascade. Thus, age-associated immune remodeling may reshape the osteoimmune microenvironment from tissue homeostasis toward chronic inflammation and degenerative remodeling through aberrant immune–structural crosstalk and mutually reinforcing feedback loops.

This communication-level remodeling provides the mechanistic link between altered immune-cell states and persistent tissue injury, because pathological inputs are repeatedly transmitted across tissue compartments while mechanisms responsible for debris clearance, inflammatory termination, and repair remain insufficiently restored.

**Figure 2** summarizes the major intercellular communication axes discussed below. **Table 3** summarizes selected immune–tissue crosstalk axes relevant to age-associated remodeling in OA; these pathways should not all be interpreted as directly caused by immunosenescence.

**Figure 2:**
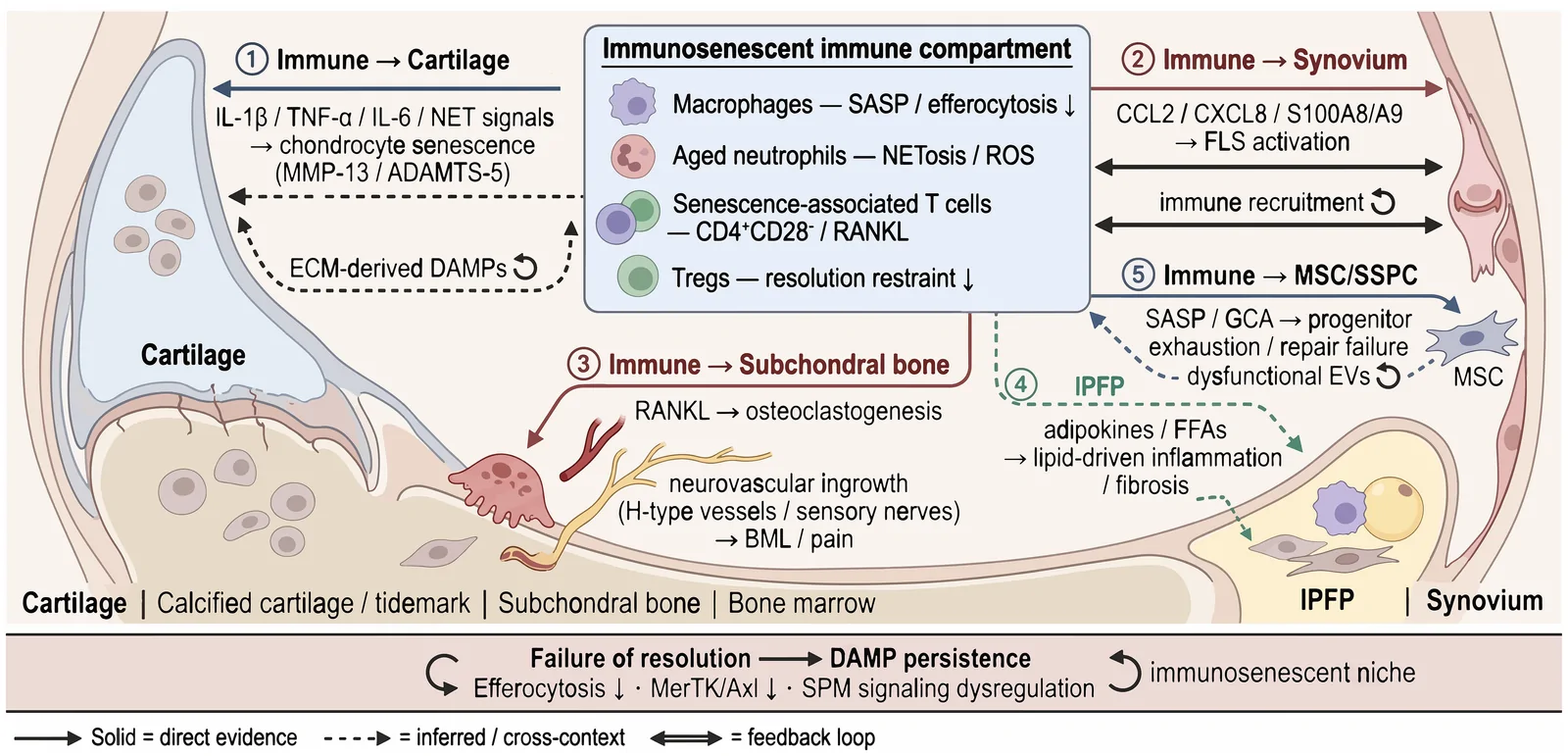
Whole-joint osteoimmune crosstalk map: immunosenescence reshapes intercellular communication in OA.

**Table 3:** Selected immune–tissue crosstalk axes in the age-remodeled OA osteoimmune microenvironment.

Crosstalk axis	Primary signaling messengers	Receptor & downstream pathway	Pathological outcome in OA	Ref.
Immune–Cartilage	IL-6, TNF-α, MMP-13, Exosomal microRNAs	TNFR1/2, IL-6R →NF-κB/STAT3	Chondrocyte senescence, Aggrecan & Collagen II degradation	(66–76)
Immune–Synovium	ANGPTL4, CCL2, CXCL8, CXCL12	α5β1 integrin, CCR2, CXCR2, TLR4 →p38 MAPK	Senescent SIF-mediated macrophage recruitment and pro-inflammatory polarization; synovial fibroblast activation and hyperplasia	(78–85)
Immune–Subchondral Bone	RANKL, OPG deficit, TNF-α	RANKL–RANK–NFATc1 axis; altered Wnt/β-catenin signaling	Uncoupled bone remodeling, BMLs, aberrant H-type angiogenesis	(87–91, 95–101)
Immune–IPFP	Leptin, adiponectin, free fatty acids, senescence-associated signals	Ob-R; TLR4–NLRP3 axis (putative)	IPFP adipocyte/stromal remodeling, immune-cell infiltration and fibrosis; senescence-associated contribution remains emerging	(102–114)
Immune–MSC	SASP factors, TGF-β, ROS	PLXNB2–Arg2 (fracture-healing model; OA relevance untested)	MSC/SSPC dysfunction, impaired cartilage repair, and altered osteogenic/adipogenic potential,	(115–121)

### 3.1 Immune–cartilage crosstalk

Avascular articular cartilage depends extensively on paracrine cues transmitted through the synovial fluid and adjacent joint compartments. In the aging joint, inflammatory macrophages and hyper-reactive neutrophils act as prominent paracrine drivers, altering chondrocyte physiological fitness.

#### 3.1.1 Inflammation-induced chondrocyte stress and senescence-associated remodeling

Macrophage-derived IL-1β, TNF-α, and IL-6 promote inflammatory activation of chondrocytes, shifting them toward catabolic states (66, 67). NET-derived DNA, histones, proteases, and oxidants add oxidative and genotoxic stress, promoting senescence-associated remodeling (68).

#### 3.1.2 Inflammatory and senescence-associated signaling drives matrix degradation

Senescent or cytokine-stimulated chondrocytes reduce type II collagen and aggrecan synthesis and increase MMP-13 and ADAMTS-5, providing the principal cartilage-specific structural consequence of immune-derived stress (66, 69).

#### 3.1.3 Vicious degradation–inflammation feedback loop

Cartilage breakdown releases ECM-derived DAMPs that stimulate pattern-recognition receptors on chondrocytes and synovial immune cells, thereby feeding inflammatory signaling back into the cartilage compartment (70–72). The distinctive feature of this axis is therefore a degradation–inflammation feedback loop in which matrix breakdown becomes a source of renewed immune activation.

#### 3.1.4 Extracellular vesicle-mediated communication

EVs provide a complementary, context-dependent communication route. Human OA cartilage-derived cells show that senescent chondrocyte EVs can transfer senescence-associated phenotypes and suppress anabolic functions *in vitro (*73). Naturally aged mice further show that aged bone marrow macrophage EVs can propagate paracrine senescence (74), while OA synovial macrophage EVs carrying miR-155-5p impair GSK-3β/mTORC1-mediated autophagy in chondrocytes (75).

EV effects are source-, state-, and cargo-dependent rather than uniformly pathogenic. Apoptotic bodies derived from M2 macrophages, for example, can promote a reparative phenotype through miR-21-5p and alleviate OA-associated inflammation and cartilage destruction (76).

Overall, EV signaling is best viewed as a dynamic communication mechanism whose biological effect depends on cellular source and state. Together with insufficient clearance of apoptotic cells and matrix debris, this context dependence allows cartilage to shift from a signal recipient to an active amplifier of joint inflammation.

### 3.2 Immune–synovium crosstalk

The synovium represents a vital immunological interface regulating joint homeostasis. Under physiological conditions, macrophage-like and fibroblast-like synoviocytes (FLS) maintain synovial fluid lubrication, tissue barrier integrity, and local immune surveillance. In OA, the synovium undergoes lining hyperplasia, immune cell infiltration, sublining fibrosis, and neoangiogenesis, transitioning from a homeostatic barrier into a persistent source of inflammatory and catabolic mediators (77).

Recent spatially and single-cell–resolved analyses of the OA synovium have begun to localize senescent stromal cells within defined synovial compartments. In surgically induced (DMM) murine OA models, senescent cells within the OA synovium were found to reside predominantly in the synovial intimal fibroblast (SIF) compartment, in which the transcription factors EGR1 and ATF3 mediate senescence induction. Functionally, senescent SIF promoted macrophage polarization toward a pro-inflammatory M1-like state and induced OA-like phenotypic changes in chondrocytes through angiopoietin-like 4 (ANGPTL4) signaling via α5β1 integrin on recipient cells, thereby establishing a direct, senescence-driven communication axis linking the synovial stroma to both the immune and cartilage compartments (78).

#### 3.2.1 Inflammatory activation and senescence-like remodeling of FLS

FLS exposed to inflammatory and metabolic stress acquire catabolic and senescence-associated features (79). Nevertheless, human OA-FLS and experimental OA models indicate that metabolic and inflammatory stress can promote senescence-associated remodeling of FLS. For example, impaired autophagy in FLS from OA patients was associated with increased senescence and SASP expression, whereas experimental restoration of autophagy reduced the senescent phenotype (80). In another study, lactate exposure promoted senescence of human OA-FLS through the ARG2–mTOR/S6K1 axis, accompanied by increased ROS, G1/S arrest, and SASP production; concordant findings were observed in rat OA and intra-articular lactate models (81). These findings support a reciprocal relationship in which the inflammatory and metabolically altered OA niche can induce FLS senescence, while senescent FLS may subsequently reinforce local inflammatory signaling.

#### 3.2.2 Chemokine-mediated immune-cell recruitment

FLS and synovial macrophages produce CCL2, CCL3, CXCL8, and CXCL12, sustaining leukocyte recruitment and retention together with endothelial activation (82). In the OA synovium, these chemotactic signals act in concert with increased vascular permeability, endothelial activation, and local inflammatory cues to sustain immune cell influx. Notably, OA synovial macrophages do not represent a rigid “M1/M2” binary, but comprise multiple subsets displaying distinct inflammatory, barrier-forming, fibrotic, and metabolic profiles; thus, the chemokine network likely shapes the OA synovial niche by modulating dynamic cellular states rather than simply driving the accumulation of a single pro-inflammatory subtype (83).

#### 3.2.3 Reciprocal remodeling of infiltrating immune cells

After recruitment, immune cells are further conditioned by the synovial metabolic and cytokine milieu, reinforcing inflammatory and tissue-remodeling states. Although this environment may favor senescence-associated or dysfunctional phenotypes, evidence remains insufficient to classify all infiltrating OA immune cells as classically immunosenescent (84).

#### 3.2.4 Synovial hyperplasia and self-sustaining inflammation

The bidirectional crosstalk between age-associated immune alterations and abnormal FLS activation promotes synovial lining hyperplasia, sublining fibrosis, and angiogenesis. FLS and macrophage-derived VEGF and FGF support endothelial activation and leukocyte entry, reinforcing the inflammatory circuit (77).

Matrix turnover and tissue injury further generate debris that feeds back to synovial cells. Together with immune recruitment, stromal activation, and angiogenesis, this debris accumulation establishes a self-reinforcing amplification circuit that challenges the capacity of resident macrophages to terminate inflammation (85). Thus, senescence-associated stromal states and immune-cell remodeling may increase the intensity and persistence of immune–synovial communication, linking this axis to the resolution bottleneck formalized in the Resolution Failure Hypothesis.

### 3.3 Immune–subchondral bone crosstalk

The subchondral bone consists of the subchondral bone plate and underlying trabecular bone marrow, forming a tightly coupled functional unit with articular cartilage via the osteochondral interface. This region not only transmits mechanical loads but also mediates bidirectional osteochondral communication through cytokines, growth factors, vasculature, and neuronal networks. Subchondral bone in OA is not merely a passive recipient of cartilage degeneration, but exhibits prominent bone remodeling, angiogenesis, immune alterations, and sensory nerve ingrowth that actively contribute to disease progression (86).

#### 3.3.1 RANKL/OPG dysregulation and osteoclast-mediated remodeling

The RANKL/OPG/RANK axis is a master regulator of osteoclastogenesis; RANK activation induces NFATc1 and terminal osteoclast differentiation (87, 88).

In OA subchondral bone, altered RANKL signaling and osteoclast activity correlate with aberrant bone remodeling and disease progression. Recent studies indicate that RANKL-mediated early subchondral bone resorption can precede overt histological cartilage degeneration, suggesting that aberrant bone remodeling participates in early OA pathogenesis (89). Immune cells may also contribute to this process: activated T cells and macrophages express RANKL, and senescence-associated CD4^+^CD28^-^ T cells in other osteolytic diseases exhibit elevated RANKL expression and promote osteoclast differentiation, suggesting that immunosenescent T-cell phenotypes may enhance local osteoclastogenesis (90).

However, current evidence is insufficient to attribute RANKL/OPG imbalance in OA entirely to senescent immune cells, as osteocytes and osteoblasts remain primary local regulators. In this regard, analyses of osteoblasts isolated from human osteoarthritic subchondral bone have shown that the differential expression of OPG and RANKL in these cells mirrors their altered metabolic and disease state, providing direct human evidence that stromal-derived shifts in the RANKL/OPG balance contribute independently to uncoupled remodeling (91). Thus, immunosenescence more likely amplifies, rather than independently initiates, RANKL-dependent aberrant bone remodeling by modifying the inflammatory and intercellular signaling milieu.

#### 3.3.2 Bone marrow lesions and uncoupled remodeling

Bone marrow lesions (BMLs) represent a prominent MRI hallmark of subchondral bone in OA, typically identified as high-signal-intensity regions on fat-suppressed T2-weighted or STIR sequences. Histological studies show that BMLs do not represent a single pathological entity, but encompass bone marrow edema-like changes, fibrosis, osteonecrosis, trabecular microdamage, and abnormal bone turnover (92). BMLs correlate with knee OA pain and structural progression, reflecting sustained mechanical loading, microdamage repair, and localized bone metabolic disturbances (93).

Within BMLs, mechanical stress and osteocyte strain interact with inflammatory inputs to promote osteoclast activity, angiogenesis, and abnormal osteogenesis. OA bone remodeling is also phase-dependent: early resorption may give way to formation and sclerosis, with Wnt/β-catenin contributing to abnormal osteogenesis (94–96).

However, the precise role of Wnt/β-catenin in OA subchondral bone remodeling exhibits marked spatiotemporal and context dependency, and direct evidence linking specific Wnt alterations to immunosenescence remains limited. Therefore, osteoimmune imbalance in OA subchondral bone is more appropriately characterized as spatiotemporally dysregulated bone turnover and remodeling rather than continuous unidirectional bone resorption (97).

Immunosenescence may further amplify this uncoupled remodeling through sustained pro-inflammatory signaling. Inflammatory mediators produced by senescence-associated immune cells and bone marrow stromal cells can disturb paracrine communication among osteoclasts, osteoblasts, and osteocytes, promoting uncoupling between bone resorption, repair, and mineralization. However, because direct evidence that BMLs represent local aggregates of senescent immune cells is lacking, BMLs are best regarded as mechanically and immunologically altered subchondral niches.

#### 3.3.3 Angiogenic remodeling and neurovascular ingrowth

Type-H vessel expansion couples angiogenesis with osteogenesis, with VEGF, PDGF-BB, TGF-β, and RANKL/RANK contributing to the osteochondral remodeling network (98). Notably, these pro-angiogenic factors originate not solely from immune cells, but also from pre-osteoclasts, osteoblasts, osteocytes, and other bone marrow stromal cells. Thus, within the context of immunosenescence, chronic inflammation and the senescent secretome more plausibly alter the local angiogenic signaling network, amplifying aberrant vessel–bone formation coupling.

Abnormal vessels can also extend through osteochondral channels into calcified cartilage and superficial layers, accompanied by sensory nerve ingrowth. In OA patients and animal models, aberrant growth of blood vessels and sensory nerves occurs at the osteochondral junction, where an increase in CGRP-positive sensory nerve fibers correlates with OA pain (99). NGF/TrkA signaling represents a major regulatory axis in this process, and attenuating NGF signaling or blocking TrkA reduces aberrant nerve ingrowth and alleviates OA pain behaviors (100).

Furthermore, osteoclasts can directly participate in sensory innervation. Studies demonstrate that subchondral bone osteoclasts secrete netrin-1, which promotes sensory nerve axonal growth into subchondral bone via DCC signaling; inhibiting RANKL-mediated osteoclastogenesis or specifically silencing osteoclast-derived netrin-1 reduces aberrant nerve ingrowth and OA pain behavior (101).

Within the subchondral compartment, immunosenescence is therefore more appropriately viewed as an amplifier of an already dysregulated remodeling environment than as the sole initiating mechanism. Persistent immune-derived inflammatory signals may interfere with coordinated osteoclast–osteoblast–osteocyte communication and delay restoration after microdamage, thereby linking chronic inflammatory persistence with abnormal bone turnover without implying that immunosenescence alone explains BML formation or osteochondral remodeling.

### 3.4 Immune–IPFP crosstalk

The IPFP is an intra-articular, extrasynovial adipose depot with active metabolic, endocrine, and immune functions. Its proximity to synovium, cartilage, and subchondral bone enables adipokines, lipid mediators, and stromal signals to influence the whole-joint environment (102, 103).

Importantly, the contribution of cellular senescence to IPFP dysfunction remains less well established than that of aging-associated tissue remodeling, obesity, or local inflammation. Previous reviews have noted the presence of senescence-associated markers such as p16INK4a and p21 in the IPFP and proposed that aging-related accumulation of senescent cells may contribute to local inflammatory remodeling; however, the tissue-specific causal role of these cells in OA has not been fully defined (104). In a human comparative study designed to separate aging from joint pathology, IPFP samples from healthy young patients undergoing anterior cruciate ligament reconstruction (n = 24) were compared with IPFP from elderly cadaver donors (n = 23), with groups matched for body mass index. Aging was associated with adipocyte enlargement and extracellular matrix remodeling even in the absence of histological inflammatory features, indicating that age itself can alter IPFP structure and function independently of established OA pathology (105). Thus, current evidence supports an age-associated remodeling of the IPFP, while the extent to which this remodeling is specifically driven by cellular senescence remains to be determined. Aging, mechanical stress, obesity, and chronic low-grade inflammation may therefore act as interacting rather than mutually exclusive determinants of IPFP dysfunction.

#### 3.4.1 Adipokine dysregulation and paracrine signaling

The IPFP in OA patients exhibits altered secretion and expression of adipokines and inflammatory mediators, but the magnitude and direction of these changes vary according to patient characteristics and experimental design. Human studies have compared IPFP from patients with established OA with healthy or non-OA controls. For example, Conde et al. reported differential adipokine expression in IPFP and synovium from OA patients and healthy individuals (106). In a larger study of 53 patients, Belluzzi et al. compared IPFP from patients with end-stage OA with samples obtained during anterior cruciate ligament reconstruction and found greater inflammation and vascularization, larger adipocytes, and altered adipokine-related gene expression in OA IPFP; notably, adipocyte size was correlated with body mass index within the OA group (107). Similarly, a study of 69 patients undergoing total knee replacement for end-stage OA demonstrated that obesity was associated with larger IPFP adipocytes and increased immune-cell populations within the IPFP, indicating that body mass and metabolic status can independently influence IPFP remodeling (108). These findings indicate that altered IPFP secretomes should not be attributed to senescence alone, because aging, obesity, and OA severity may contribute simultaneously. Among these, leptin displays prominent pro-catabolic actions. In an experimental study using conditioned media from osteoarthritic joint adipose tissue together with cultured chondrocytes and cartilage explants, leptin activated STAT, MAPK, Akt, and NF-κB signaling and increased MMP1 and MMP13 expression; the effects of IPFP-derived conditioned media were partially attenuated by leptin neutralization (109). These findings support an IPFP–cartilage metabolic and paracrine axis through which altered adipokine signaling can sustain a catabolic chondrocyte state and potentially reinforce secondary senescence.

#### 3.4.2 Lipid dysregulation and immunometabolic signaling

The IPFP also releases free fatty acids and derived lipid mediators. Human ex vivo lipidomic studies have demonstrated disease-associated alterations in the lipid profile of the IPFP. Gierman et al. analyzed conditioned media from IPFP explants obtained from 13 patients with end-stage OA undergoing joint replacement and 10 normal donors obtained postmortem, with an additional validation set of 23 OA donors, and identified distinct profiles of fatty acids and oxylipins between OA and normal samples (110). Similarly, Mustonen et al. compared paired IPFP and synovial-fluid samples from 10 OA and 10 rheumatoid arthritis patients undergoing total joint replacement, with six control synovial-fluid samples from individuals without inflammatory or degenerative knee disease, and demonstrated disease-specific alterations in fatty-acid composition (111). These studies provide evidence that lipid metabolism in the IPFP is altered in joint disease, but they were not designed to isolate the effects of cellular senescence from aging, disease stage, or other metabolic factors.

In general adipose tissue and macrophages, saturated fatty acids—particularly palmitate—can amplify NF-κB and NLRP3 inflammasome activation via TLR-associated signaling, endoplasmic reticulum stress, ROS generation, and ceramide accumulation (112). While analogous lipid–immunometabolic mechanisms may operate within the IPFP, direct evidence demonstrating that IPFP-derived saturated fatty acids directly drive adjacent tissue inflammation via the TLR4–NLRP3 axis in human OA remains limited. Thus, this mechanism currently serves as a plausible hypothesis linking IPFP senescence, lipid dysregulation, and immune activation rather than an established OA pathway.

#### 3.4.3 Macrophage–adipocyte interactions and IPFP fibrosis

Macrophages in the IPFP establish an intimate local communication network with adipocytes and stromal cells. In the OA IPFP, macrophage infiltration and pro-inflammatory polarization are readily observed; these macrophages secrete IL-6, TNF-α, and other mediators that interact with adipocytes to shape the local inflammatory environment. Concurrently, the OA IPFP undergoes marked fibrosis and vascular remodeling. Recent single-cell transcriptomic studies have revealed that mesenchymal lineage cells within the IPFP shift toward inflammatory and pro-fibrotic phenotypes, with specific inflammatory fibroblast subsets capable of modulating macrophage and T-cell functions (113, 114). Therefore, IPFP fibrosis may represent a key intersection between immunosenescence and stromal remodeling. Sustained inflammatory signaling, TGF-β-dependent pathways, and senescence-associated secretomes can stimulate fibroblast activation and ECM deposition, altering IPFP tissue architecture and biomechanical properties. However, this causal sequence remains largely inferential and requires rigorous mechanistic validation.

Thus, altered immune states may remodel the IPFP not simply by increasing inflammatory cell numbers, but by altering the functional state of adipocytes, macrophages, and mesenchymal stromal cells; the resulting shifts in adipokine secretion, lipid metabolism, inflammatory signaling, and extracellular matrix remodeling position the IPFP as a metabolic and inflammatory amplifier within the whole-joint network, increasing the inflammatory load imposed on the synovium, cartilage, and subchondral bone. However, because these alterations reflect the combined influence of aging, obesity, OA severity, mechanical stress, and local inflammation, and direct evidence isolating the contribution of IPFP cellular senescence remains limited, senescence should presently be regarded as a potentially important modifier of IPFP–immune crosstalk rather than an established sole driver of IPFP dysfunction. This distinction is important: IPFP remodeling may feed into impaired whole-joint homeostasis without constituting a direct or exclusive manifestation of immunosenescence.

### 3.5 Immune–MSC crosstalk

MSCs and skeletal stem/progenitor cells (SSPCs) reside within specialized niches such as bone marrow, synovium, and the IPFP, serving as vital cellular reservoirs for tissue homeostasis and post-injury repair. Beyond their multilineage osteogenic and chondrogenic differentiation potential, MSCs exert prominent immunomodulatory functions, regulating macrophages, dendritic cells, and T-cell populations through paracrine mediators and EVs. Consequently, the functional decline of MSCs/SSPCs can concurrently compromise tissue regeneration and inflammation resolution (115).

#### 3.5.1 SASP-driven progenitor dysfunction and secondary senescence

In aging and chronic inflammatory milieus, persistent inflammatory and senescence-associated cues impair MSC/SSPC self-renewal and tissue repair capacities. Studies on joint-derived MSCs from OA patients demonstrate that prolonged exposure to senescence-associated inflammatory factors induces cell-cycle arrest and reduces MSC self-renewal, suggesting that the senescent microenvironment reshapes neighboring MSC cellular states via paracrine mechanisms (116).

Evidence from skeletal regeneration provides a mechanistic analogy rather than direct OA validation: in a fracture-healing model, Zou et al. reported that grancalcin (GCA) secreted by senescence-associated macrophages induced mitochondrial dysfunction and cellular senescence in SSPCs through the PLXNB2–Arg2 axis, reducing osteogenic and chondrogenic potential and delaying fracture healing (117). Whether this axis operates in OA joint-resident progenitors remains to be determined.

#### 3.5.2 Age-associated lineage bias and regenerative decline

Senescence in MSCs and SSPCs also manifests as alterations in multilineage differentiation potential and regenerative capacity. With advancing age, skeletal stem cells display diminished osteogenic and chondrogenic capacities and yield more stromal progeny with pro-inflammatory and pro-resorptive phenotypes, further remodeling the bone marrow niche (118). In OA-associated MSCs, senescence similarly correlates with reduced proliferation, impaired chondrogenic differentiation, and elevated cellular senescence markers (119). While MSCs from certain tissue sources exhibit an adipogenic-to-osteogenic/chondrogenic lineage skewing, such alterations are highly tissue- and niche-dependent and should not be generalized across all senescent MSC populations.

#### 3.5.3 Loss of immunoregulatory fitness and dysfunctional extracellular vesicle signaling

MSCs function not only as regenerative progenitors but also as crucial immunoregulatory cells. Healthy MSCs suppress pro-inflammatory T-cell and macrophage responses while fostering immunotolerant phenotypes, thereby restraining persistent tissue inflammation (120). However, intrinsic senescence of MSCs impairs this immunomodulatory competence and alters their paracrine signaling repertoire.

Notably, MSC-derived EVs represent an important conduit linking MSC senescence to joint inflammation. Recent studies demonstrate that EVs derived from senescent human adipose-derived MSCs exhibit a diminished capacity to suppress pro-inflammatory macrophage polarization and lose the cartilage- and subchondral bone-protective effects characteristic of normal MSC-EVs in OA models; in some instances, senescent MSC-EVs can even transiently induce pro-catabolic and pro-inflammatory gene expression (121). These findings indicate that MSC senescence entails not only a loss of regenerative fitness, but also a qualitative shift in intercellular communication from tissue-protective paracrine signaling toward a dysfunctional secretome.

#### 3.5.4 Summary: consequences of osteoimmune microenvironmental remodeling

The five osteoimmune compartments examined above should not be interpreted as independent pathological units. Rather, they form a connected network in which altered immune-cell states generate persistent inflammatory, metabolic, and damage-associated signals; structural and stromal cells respond with stress, senescence-associated, catabolic, or fibrotic phenotypes; and the resulting tissue-derived signals further reinforce immune activation. Across these compartments, the dominant mechanisms differ: cartilage is characterized by matrix degradation and DAMP-mediated feedback, the synovium by reciprocal stromal–immune activation, the subchondral bone by dysregulated remodeling and osteochondral signaling, the IPFP by metabolic and inflammatory amplification, and MSC/SSPC niches by impaired regenerative competence.

Importantly, these compartment-specific processes converge on a common systems-level problem: persistent inflammatory input is not adequately matched by endogenous mechanisms of inflammatory termination, apoptotic-cell clearance, and tissue repair. Impaired efferocytosis provides direct human OA evidence for defective clearance capacity, whereas abnormalities in MerTK/Axl signaling, SPM activity, and senescence-associated resolution mechanisms are supported to varying degrees by experimental OA, aging, and other disease models. Thus, the available evidence supports resolution deficiency as a plausible mechanistic bridge connecting immunosenescence-associated cellular remodeling with persistent whole-joint pathology, while the extent to which this process is causal, rather than secondary to established tissue injury and degeneration, remains to be determined.

This framework shifts the interpretation of OA from a collection of compartment-specific inflammatory pathways toward a coupled sequence in which age-associated immune remodeling alters intercellular communication, persistent signaling overwhelms or impairs resolution capacity, and incomplete tissue restoration feeds back into further structural damage. The therapeutic implication is correspondingly broader than senescent-cell elimination alone: durable microenvironmental reprogramming may require coordinated reduction of pathogenic senescence-associated signals together with restoration of inflammatory resolution and regenerative competence.

## 4 Therapeutic reprogramming of the immunosenescent microenvironment

Halting OA progression necessitates a fundamental conceptual paradigm shift from conventional “symptom suppression” toward microenvironmental “therapeutic reprogramming.” Standard NSAIDs and single-cytokine biologics have generally shown limited ability to prevent structural joint deterioration, while leaving many of the interconnected biological processes underlying OA insufficiently addressed. Moreover, clinical trials of several single-pathway anti-inflammatory or biological therapies have generally failed to demonstrate sufficient disease-modifying effects, highlighting the biological heterogeneity and multifactorial nature of OA (13, 122). Within this context, therapeutic reprogramming of the immunosenescent microenvironment represents a complementary strategy that seeks to target multiple interconnected pathological processes rather than a single inflammatory mediator.

Therapeutic reprogramming aims to attenuate pathological intercellular signaling, suppress persistent SASP-associated programs, and restore metabolic flexibility and efferocytic competence across the osteoimmune microenvironment. By targeting senescent-cell survival mechanisms, modulating nutrient-sensing pathways, and implementing biomaterial-assisted interventions, this strategy seeks to shift the local microenvironment toward inflammatory resolution and tissue repair.

**Figure 3** integrates the therapeutic strategies discussed in this section into a stage- and mechanism-oriented reprogramming framework. Rather than representing a fixed drug sequence, the four-step workflow progresses from selective elimination or silencing of pathogenic senescence-associated signals to restoration of metabolic and resolution capacity and, ultimately, reconstruction of a regenerative niche. The relative contribution of each module is expected to depend on disease stage, tissue compartment, and the dominant biological endotype. **Table 4** outlines representative therapeutic strategies for reprogramming the immunosenescent osteoimmune microenvironment.

**Figure 3:**
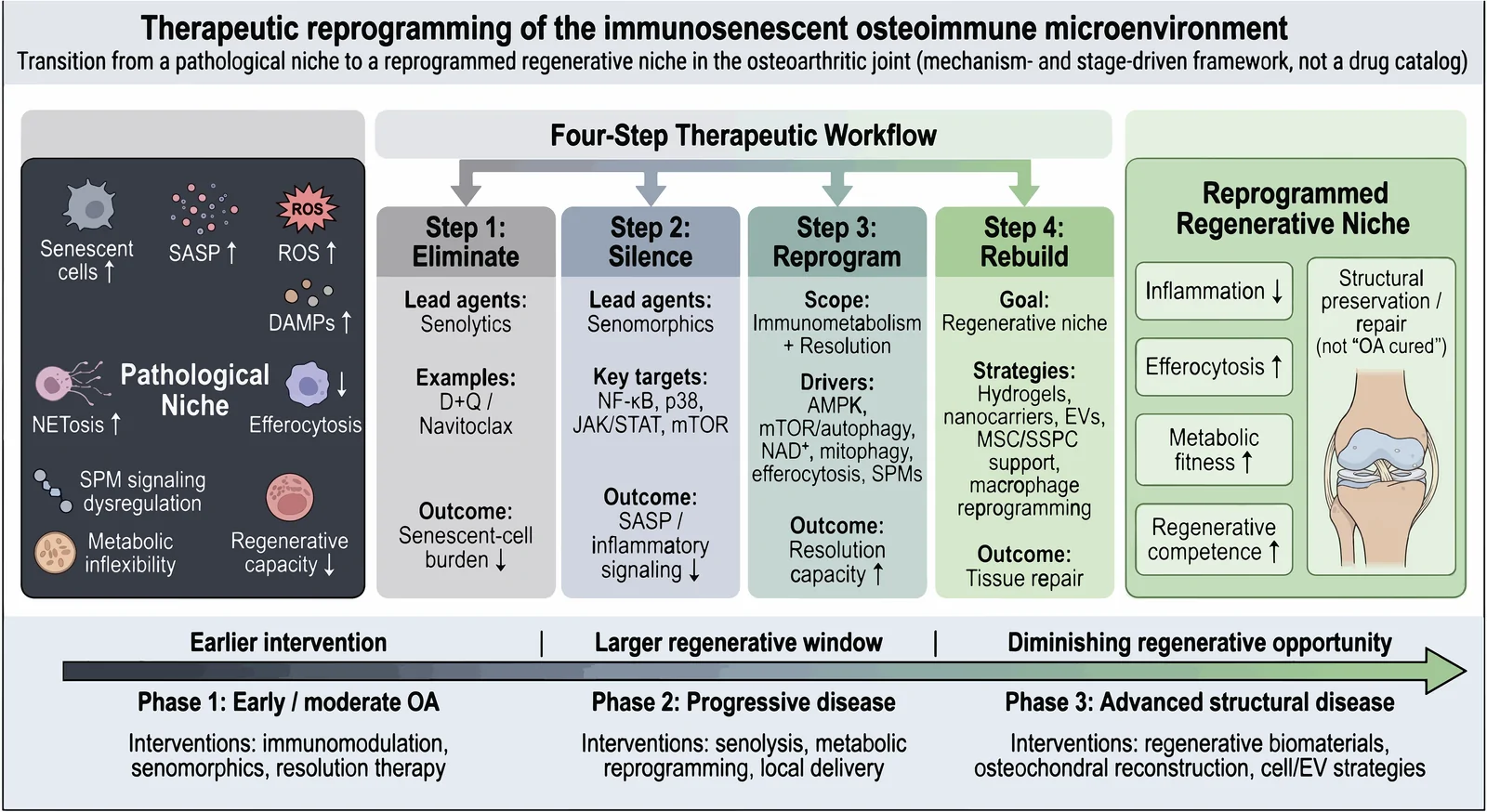
Therapeutic reprogramming of the immunosenescent osteoimmune microenvironment.

**Table 4:** Therapeutic strategies for reprogramming the immunosenescent osteoimmune microenvironment.

Therapeutic category	Target/lead agents	Mechanism of action	Translational stage & limitations	Ref.
Senolytics	D+Q; Navitoclax (ABT-263)	SCAP disruption (BCL-2/BCL-xL, PI3K/AKT) → selective senescent-cell apoptosis	Preclinical/early trials; analgesic without consistent structural benefit	(14–17)
Senomorphics	NF-κB/p38 & JAK inhibitors; Rapamycin; Metformin; Pterostilbene	SASP transcriptional silencing without cell elimination	Preclinical; durable human disease modification unproven	(130–136)
Immunometabolic Reprogramming	Metformin (AMPK); Rapamycin (mTORC1); NMN/NR; CD38 inhibitors	Restore metabolic flexibility, autophagy/mitophagy; reduce mtROS–mtDNA signaling	Preclinical; cell-type- and context-dependent effects	(138–144)
Resolution-oriented therapy	MerTK/Axl modulators; SPMs (RvD1, MaR1); pCQ/SOD nanoparticles	Restore efferocytosis; active inflammation termination	Preclinical/early clinical (GAUDI); intra-articular evidence limited	(145–152, 184)
Biomaterial-assisted delivery	D+Q-loaded targeted liposomes; ABT263-loaded lesion-localized hydrogels; senescence-targeted si-ADAM19 nanoparticles; mitochondria-/ROS-responsive hydrogels; bioprinted scaffolds	Cell-specific senolysis; senescence-associated pathway modulation; redox/mitochondrial restoration; spatiotemporal therapeutic release	Preclinical; limited/no human validation; targeting specificity, biodegradability, safety, scalability, and long-term tissue integration remain concerns	(126–129, 153–158)
Cell & secretome rejuvenation	Hypoxia-preconditioned MSCs; miR-140 EVs; engineered MSC/EV platforms	Paracrine immunomodulation; targeted cargo; efferocytosis restoration	Preclinical; GMP manufacturing and regulatory barriers	(161–165)

### 4.1 Eliminating pathogenic senescent niche cells

Current OA therapies remain largely focused on symptom control and modulation of individual inflammatory pathways, while their ability to modify the interconnected biological processes underlying structural progression is limited. Growing evidence that cellular senescence and immunosenescence contribute to persistent inflammation, matrix degradation, and impaired tissue repair has therefore motivated the development of senotherapeutic and microenvironment-directed strategies.

#### 4.1.1 Mechanisms and major targets

Senolytics are designed to transiently disrupt survival pathways on which selected senescent cell populations depend, thereby promoting their apoptosis while minimizing effects on nonsenescent cells. Established senolytic targets include BCL-2 family proteins, PI3K/AKT-related survival signaling, HSP90, and other context-dependent anti-apoptotic pathways. Importantly, p16INK4a and p21CIP1 primarily represent components or markers of senescence-associated cell-cycle arrest rather than universal pharmacological targets of senolytics (14).

Dasatinib and quercetin (D+Q) and the BCL-2/BCL-xL inhibitor navitoclax (ABT263) are among the most extensively investigated senolytic strategies in musculoskeletal disease (15). In human OA cartilage explants and isolated chondrocytes, D+Q selectively reduced senescent cells, decreased SASP-associated IL-6 and CXCL1 release, and promoted the expression of anabolic markers including COL2A1, ACAN, and SOX9. Similarly, ABT263 has been shown to reduce senescent chondrocytes, attenuate inflammatory signaling, and improve cartilage and subchondral bone pathology in experimental post-traumatic OA, including following intra-articular administration (16, 17).

#### 4.1.2 Microenvironmental consequences

The therapeutic rationale of senolysis extends beyond simply reducing senescent-cell numbers. By removing selected SASP-producing cells, senolytics may decrease the local concentration of inflammatory cytokines and matrix-degrading mediators, thereby reducing secondary senescence and creating a more permissive environment for endogenous tissue repair (14). In human OA chondrocytes, D+Q-induced senolysis was accompanied by a shift toward a more chondroanabolic phenotype, suggesting that removal of senescent cells may partially restore local tissue signaling rather than merely suppress inflammation (15).

However, the disease-modifying potential of senolytics should not be overstated. In aged mice with spontaneous OA, systemic treatment with ABT263 or D+Q reduced peripheral nociceptive signaling and OA-associated pain but did not consistently modify cartilage degeneration or abnormal subchondral bone changes (18). These findings suggest that senolytic efficacy may depend strongly on the OA phenotype, disease stage, senescent-cell composition, and tissue distribution of the targeted cells.

#### 4.1.3 Selectivity and temporal considerations

An important challenge is that cellular senescence is not intrinsically pathological. Transient senescence can participate in tissue remodeling, regeneration, and acute wound repair, whereas persistent senescence is more strongly associated with chronic inflammation and tissue dysfunction (123). Therefore, indiscriminate or prolonged elimination of senescent cells may interfere with beneficial physiological functions (124).

Moreover, neither p16^INK4a^ nor p21^CIP1^ alone is sufficient to define a pathogenic or beneficial senescent state. Recent single-cell analyses have demonstrated that p16-positive and p21-positive cells frequently represent distinct populations with tissue-dependent molecular trajectories and substantially different secretory profiles (125). These findings argue against relying on a single senescence marker for therapeutic targeting and support a more context-dependent approach that considers cellular identity, tissue location, persistence, SASP composition, and functional phenotype.

#### 4.1.4 Local delivery and biomaterial-enabled targeting

For OA, local intra-articular delivery represents an attractive strategy for increasing drug exposure within the joint while potentially reducing systemic exposure. Emerging antisenescence biomaterial platforms—including nanoparticles, lipid-based carriers, EVs, hydrogels, and composite delivery systems—may further improve the spatial and temporal control of senotherapeutic interventions by enhancing local retention, targeting, and controlled release (126). Several preclinical studies have provided proof-of-concept for this approach.

One representative strategy targeted senescent FLSs. Chen et al. developed an aptamer-functionalized liposome (CX3-LS-DQ) encapsulating the senolytic combination D+Q. The system showed preferential affinity for FLSs and enhanced clearance of senescent FLSs, while intra-articular administration in an OA mouse model attenuated cartilage degradation (127). This study illustrates how cell-targeting ligands can improve the local specificity of conventional senolytics and potentially reduce off-target effects.

Hydrogel-based systems have also been developed to combine local retention with senolytic delivery. Xiong et al. constructed cartilage lesion-localized hydrogel microspheres composed of aldehyde- and methacrylic anhydride-modified hyaluronic acid, incorporating ABT263-loaded liposomes and PDGF-BB (A-Lipo/PAHM). *In vitro*, the ABT263-containing liposomes preferentially induced apoptosis of senescent chondrocytes and reduced SA-β-gal-positive cells, while the resulting apoptotic cells were subsequently cleared by macrophage efferocytosis. The microspheres also recruited endogenous mesenchymal stem cells through sustained PDGF-BB release. In cartilage explant and *in vivo* OA models, this combined strategy promoted clearance of senescent chondrocytes and improved cartilage repair, thereby linking senolysis with restoration of the local regenerative microenvironment (128).

Beyond direct senolysis, senescence-targeting biomaterials have been explored to suppress or reverse senescent phenotypes. Wang et al. developed galacto-oligosaccharide-coated hollow mesoporous silica nanoparticles for senescence-responsive delivery of siRNA against ADAM19 (senHMSN_si-ADAM19). *In vitro*, this system preferentially released siRNA in senescent chondrocytes, reduced ADAM19 and p16INK4a expression, and restored proliferative and matrix-producing phenotypes. In a DMM-induced post-traumatic OA mouse model, intra-articular administration reduced p16-driven senescence signals, p16INK4a- and MMP13-positive cells, and synovial inflammation while promoting hyaline cartilage regeneration (129).

More recently, immuno-targeted nanoparticles have been used to directly reprogram senescent immune cells. Liu et al. developed pCQ/SOD polymeric nanoparticles to preferentially deliver superoxide dismutase to synovial macrophages. Intra-articular treatment reduced macrophage senescence, mitochondrial damage, mtDNA release, and SASP production, while enhancing efferocytosis through the STAT3/ADAM17/MerTK pathway and promoting M1-to-M2 repolarization. Importantly, the intervention alleviated both trauma-induced and aging-induced OA in mice, providing experimental evidence that biomaterial-assisted targeting of senescent immune cells can modify the joint microenvironment rather than simply suppressing inflammatory mediators (6).

Collectively, these studies indicate that biomaterial-assisted senotherapeutic strategies can intervene at several levels, including selective clearance of senescent cells, suppression of senescence-associated signaling, restoration of efferocytosis, and promotion of endogenous tissue repair. However, most current evidence remains preclinical, and differences in senescent-cell populations, OA models, treatment timing, and senolytic sensitivity may substantially influence therapeutic efficacy.

### 4.2 Silencing pathological communication networks

When broad cell elimination risks disrupting the physiological roles of transient senescence in tissue repair and homeostasis, suppressing aberrant paracrine communication offers a complementary approach to senolytic therapy. Distinct from directly inducing senescent cell apoptosis, senomorphic interventions aim to suppress or remodel the SASP and its downstream cascades while preserving cellular viability. The underlying rationale is to attenuate the bystander damage inflicted by senescent cells on surrounding tissues by repressing pro-inflammatory transcriptional programs, metabolic stress, and aberrant survival signaling, without requiring the complete clearance of specific senescent populations (130, 131).

#### 4.2.1 Mechanisms and major targets

SASP expression is orchestrated by multiple interconnected signaling networks, several of which have received preclinical validation in OA models. Among these, NF-κB, p38 MAPK, JAK/STAT, and mTOR-related pathways represent primary therapeutic targets. NF-κB serves as a master transcriptional regulator for diverse SASP inflammatory cytokines, while p38 MAPK cooperates with NF-κB and other stress-activated cascades to maintain senescence-associated secretory programs (132, 133). Conversely, mTOR inhibition can attenuate senescence phenotypes by restoring autophagy and metabolic homeostasis (134), while JAK/STAT signaling mediates persistent tissue responses driven by IL-6 and related inflammatory mediators (135).

#### 4.2.2 Microenvironmental effects

Through the inhibition of NF-κB, p38 MAPK, JAK/STAT, or mTOR-related signaling, senomorphic interventions may reduce selected SASP-associated cytokines and matrix-degrading mediators, including IL-6, IL-1β, MMP13, and ADAMTS5, depending on the targeted pathway and cellular context. In turn, attenuation of these mediators may decrease inflammatory recruitment, matrix catabolism, and secondary stress responses in neighboring chondrocytes, synovial cells, and other joint-resident populations (13).

Importantly, senomorphic therapy differs conceptually from conventional cytokine blockade because its therapeutic goal is not simply to neutralize one extracellular mediator, but to reshape the signaling state of senescent or chronically activated cells and thereby interrupt broader pathological communication networks. Recent reviews of OA senotherapeutics identify rapamycin, metformin, pterostilbene, and related metabolic or signaling modulators as emerging senomorphic strategies, although most evidence remains preclinical and their ability to produce durable disease modification in human OA remains uncertain (136, 137).

### 4.3 Restoring immunometabolic plasticity and resolution capacity

Beyond suppressing pro-inflammatory signals and eliminating pathogenic senescent cells, long-term microenvironmental reprogramming requires restoring metabolic adaptability, organelle quality control, and endogenous inflammation-resolution mechanisms. Cells within aging and chronic inflammatory niches exhibit metabolic rigidity, mitochondrial dysfunction, defective autophagy and debris clearance, and impaired resolution programs. Re-establishing these fundamental cellular processes is therefore essential for shifting the microenvironment from persistent inflammation back toward homeostasis and repair (11, 12).

#### 4.3.1 Nutrient sensing and metabolic reprogramming

##### 4.3.1.1 AMPK activation

AMPK functions as a central sensor coupling cellular energetic status to inflammatory and metabolic responses. In OA, metformin activates AMPK and inhibits NF-κB signaling, attenuating IL-1β-induced chondrocyte injury; in DMM and other murine OA models, metformin limits cartilage degeneration in an AMPK-dependent manner, accompanied by mTORC1 suppression (138, 139). AMPK activation may also enhance macrophage metabolic fitness and support inflammation resolution; however, its specific effects on macrophage oxidative metabolism and fatty acid oxidation (FAO) are highly cell- and context-dependent and should not be simplified to a fixed “M2 metabolic profile.”

##### 4.3.1.2 mTOR regulation and autophagy restoration

Sustained mTORC1 hyperactivation suppresses autophagy while driving cell growth and protein translation, correlating with metabolic imbalance and senescence-associated alterations in OA chondrocytes. Seminal studies demonstrate that rapamycin-mediated mTOR inhibition and autophagy induction attenuate experimental OA, while metformin similarly mitigates cartilage degeneration via the AMPK/mTOR axis (140). Furthermore, SIRT3 overexpression inhibits PI3K/AKT/mTOR signaling, restoring impaired autophagic flux and improving mitochondrial function (141). Thus, finely tuned mTORC1 inhibition and autophagy restoration represent a critical therapeutic nexus linking metabolic reprogramming to organelle quality control.

##### 4.3.1.3 Restoring NAD^+^ homeostasis

NAD^+^ metabolism is a vital axis bridging aging, mitochondrial homeostasis, and cellular stress responses. Recent studies reveal depleted NAD^+^ levels and disrupted NAD^+^ metabolic networks in human, murine, and rat OA cartilage; boosting NAD^+^ bioavailability via NMN or NR ameliorates OA-associated structural pathology in post-traumatic and age-related models, indicating that NAD^+^ deficiency actively participates in OA pathogenesis rather than being a mere bystander (142). Concurrently, CD38, a primary mammalian NADase, is upregulated in OA cartilage, and pharmacological inhibition of CD38 preserves cartilage homeostasis, mitigates subchondral bone abnormalities, and alleviates pain in joint injury models (143). Replenishing NAD^+^ may further augment SIRT1/SIRT3-dependent regulatory pathways and alleviate mitochondrial stress, though specific sirtuin-dependent mechanisms warrant deeper investigation in OA.

##### 4.3.1.4 Organelle quality control and mitophagy

Mitochondrial dysfunction is a hallmark of age-associated cellular impairment; hence, restoring proper mitochondrial quality control reduces oxidative stress and maintains cellular fitness. In OA, SIRT3 alleviates chondrocyte injury by modulating mitochondrial function and autophagy, and various experimental interventions improve mitochondrial homeostasis and attenuate OA phenotypes via PINK1/Parkin-mediated mitophagy (141). However, because PINK1/Parkin-dependent mitophagy may exhibit context-dependent roles in OA (144), the therapeutic objective is not simply to maximize mitophagy indiscriminately, but rather to restore balanced and efficient mitochondrial quality control, thereby lowering mtROS, limiting mitochondrial DAMP release, and constraining cGAS–STING and NLRP3 inflammasome activation.

##### 4.3.1.5 Restoring efferocytosis and inflammation resolution

Restoring macrophage-mediated clearance of apoptotic cells represents a cornerstone for re-establishing inflammation resolution. Human studies demonstrate that knee OA synovium harbors increased numbers of apoptotic cells, while synovial macrophages exhibit a reduced efferocytic index and lower engulfment capacity per cell, establishing efferocytosis impairment as a quantifiable feature of OA microenvironmental dyshomeostasis (145). Restoring the expression and activity of apoptotic recognition receptors, such as MerTK and Axl, may enhance macrophage-mediated clearance of apoptotic cells and cellular debris, thereby reducing persistent DAMP exposure. However, direct *in vivo* evidence for intra-articular MerTK/Axl-targeted therapy in OA remains emerging, making this strategy a promising resolution-oriented approach rather than an established clinical modality.

##### 4.3.1.6 Specialized pro-resolving mediators

Complementing intracellular metabolic restoration, direct supplementation of endogenous pro-resolving signals can re-engage the coordinated sequence of “inflammation cessation—debris clearance—tissue repair.” Specialized pro-resolving mediators (SPMs)—including lipoxins, resolvins, protectins, and maresins—act through specific G-protein coupled receptors to limit neutrophil infiltration, enhance macrophage efferocytosis, and support tissue regeneration (146). In OA models, RvD1 suppresses NF-κB, p38, and JNK activation in human chondrocytes, reducing MMP-13, COX-2, and oxidative stress (147); sustained release of RvD1 also inhibits synovial macrophage pro-inflammatory polarization while promoting a reparative phenotype, attenuating progressive cartilage degradation in murine OA (148). These findings indicate that SPM-based therapeutics can promote inflammatory resolution and tissue homeostasis in OA, while their potential effects on cellular senescence represent an emerging area of investigation.

##### 4.3.1.7 Potential links between SPM signaling and cellular senescence

Although the OA studies described above did not directly evaluate canonical senescence endpoints, emerging evidence from aging and other disease models suggests that SPMs may also modulate senescence-associated pathways. In H_2_O_2_-induced dermal fibroblasts, RvD1 reduced the senescence-associated markers p16INK4a, p21CIP1, and SA-β-galactosidase activity while promoting autophagy through the miR-1299/ARG2/ARL1 axis (149). In aged mice, RvD1 treatment reduced the expression of cellular senescence- and inflammation-associated genes in splenic macrophages and attenuated age-associated splenic fibrosis (150). These findings provide direct evidence that RvD1 can influence senescence-associated molecular and phenotypic features, although they were obtained outside the OA setting.

SPM signaling may also connect senescence to impaired inflammation resolution through macrophage function. Senescent-cell conditioned medium increased MerTK cleavage and impaired macrophage efferocytosis, whereas RvD1 prevented MerTK loss and restored efferocytic activity in experimental models of aging (151). More recently, RvD1 was shown to reduce SA-β-gal activity and reshape the SASP of inflammation-induced rat cardiac fibroblasts, including reductions in IL-1β, IL-6, TNF-α, and MCP-1 (152). Together, these observations support a potential senomorphic and pro-resolving role of RvD1, involving regulation of oxidative stress, autophagy, efferocytosis, and SASP-related inflammatory signaling.

Importantly, whether SPMs directly suppress established senescence programs or reverse immunosenescence within the osteoarthritic joint remains uncertain. In particular, current OA studies have not yet established that RvD1 or other SPMs consistently reduce senescence markers such as p16INK4a, p21CIP1, or SA-β-galactosidase, or directly suppress SASP production in senescent joint-resident cells. Therefore, the therapeutic relevance of SPMs to immunosenescence in OA should currently be regarded as an emerging mechanistic link rather than an established anti-senescence effect. Their most firmly supported role remains the restoration of inflammation resolution, macrophage efferocytosis, and tissue homeostasis.

### 4.4 Rebuilding a permissive regenerative niche

Following the reduction of pathogenic senescent cell burden, suppression of aberrant inflammatory signaling, and restoration of metabolic and resolution capacities, subsequent therapeutic objectives must transition from solely blocking pathological cues toward actively reconstructing a microenvironmental niche permissive for tissue repair. In OA, this entails not only delivering bioactive agents, but also optimizing intra-articular spatiotemporal pharmacokinetics, shielding therapeutic cargo, restoring the osteochondral interface architecture, and reinstating intercellular signaling networks that sustain progenitor survival and differentiation.

#### 4.4.1 Biomaterial-assisted local delivery

Biomaterial-assisted intra-articular delivery may provide an additional means of modulating senescence-associated pathology while improving local drug retention and reducing systemic exposure. Importantly, current evidence does not yet support the view that biomaterials directly reverse immunosenescence as a systemic or tissue-wide process. Rather, emerging strategies primarily target senescent cells or senescence-associated pathways within the joint, including oxidative stress, mitochondrial dysfunction, SASP production, and aberrant immune-cell activation. In this context, biomaterials may function as local microenvironmental reprogramming platforms that complement, rather than replace, conventional senotherapeutic approaches.

#### 4.4.2 Microenvironment-responsive hydrogels

Injectable hydrogels can provide sustained intra-articular retention and controlled release of therapeutic molecules, while stimuli-responsive designs may couple drug release to pathological features of the OA microenvironment (153, 154). A recent dual-responsive GelMA/F127DA hydrogel, for example, used MMP13 activity and mechanical loading as triggers to regulate the release of TGF-β and kartogenin and showed improved cartilage regeneration in an OA model (155). Although this strategy was primarily designed to promote cartilage repair rather than directly target cellular senescence, its responsiveness to pathological matrix degradation and mechanical stress illustrates how biomaterials can modify microenvironmental factors that contribute to senescence-associated tissue dysfunction.

#### 4.4.3 Targeted nanocarriers and nanozymes

Nanoparticle-based systems may improve intra-articular retention, cargo stability, cellular targeting, and controlled release. Importantly, several recent studies suggest that their therapeutic value may extend beyond nonspecific suppression of inflammatory mediators by directly modifying senescence-associated molecular programs. For example, a mitochondria-targeted interpenetrating-network hydrogel delivered an MnO_2_-based nanozyme together with a NAD^+^ precursor to restore mitochondrial respiratory-chain function in OA chondrocytes. The intervention reduced oxidative stress, restored the NAD^+^/NADH balance, suppressed SASP production, and alleviated chondrocyte senescence while preserving cartilage integrity in an OA model (156).

A particularly relevant immuno-targeted strategy has been developed for synovial macrophages. Liu et al. engineered pCQ/SOD polymeric nanoparticles to deliver superoxide dismutase to synovial macrophages. In OA synovium, senescent macrophages exhibited enhanced SASP production, mitochondrial damage, impaired efferocytosis, and increased inflammatory polarization. pCQ/SOD treatment inhibited p53- and cellular-senescence-associated signaling, reduced mitochondrial damage and mtDNA release, decreased SASP production, restored efferocytosis through the STAT3/ADAM17/MerTK pathway, and promoted M1-to-M2 repolarization. Importantly, intra-articular administration alleviated both trauma-induced and aging-induced OA in mice (6). This study provides comparatively direct evidence that biomaterial-assisted targeting of senescent immune cells can modify senescence-associated signaling and immune-cell function rather than merely reducing inflammatory cytokines.

ROS-scavenging nanozyme systems provide another potential route for modulating senescence-associated stress. For example, a selenium nanozyme-crosslinked injectable hydrogel combining selenium nanoparticles with platelet-rich plasma reduced ROS levels, mitigated chondrocyte senescence, restored ECM homeostasis, and improved cartilage repair in an OA rat model (157). More recently, an injectable bioadhesive hydrogel containing miR-140-5p-loaded nanoparticles was developed for aged OA and was reported to downregulate senescence-associated signaling, alleviate mitochondrial oxidative stress, and attenuate chondrocyte senescence and OA progression in aged rats (158).

These studies indicate that the relationship between biomaterial therapy and immunosenescence is currently best understood as a process of local senescence-associated pathway modulation and immune–tissue microenvironmental remodeling, rather than direct reversal of systemic immunosenescence. Senolytic delivery can selectively remove pathogenic senescent cells, whereas redox, mitochondrial, SASP, and intracellular signaling interventions may attenuate senescence-associated dysfunction while preserving viable cells. However, whether these local interventions can durably restore age-related immune homeostasis across the entire joint remains insufficiently established.

#### 4.4.4 3D-bioprinted osteochondral scaffolds

Three-dimensional bioprinting provides spatial control over cells, biomaterials, and signaling factors and may therefore be particularly useful for reconstructing the heterogeneous cartilage–calcified cartilage–subchondral bone interface. Recent reviews highlight the potential of multilayer and gradient bioprinted constructs to reproduce aspects of native osteochondral architecture and guide cell differentiation (159, 160). Nevertheless, most evidence remains preclinical, and challenges including vascularization, mechanical durability, long-term integration, bioink standardization, and manufacturing scalability continue to limit clinical translation.

#### 4.4.5 Cell and secretome rejuvenation

##### 4.4.5.1 Rejuvenating MSCs and progenitor cells

MSC-based therapy faces major challenges after intra-articular administration, including poor cell retention and survival within the inflammatory and metabolically stressed OA joint. Hypoxic preconditioning can improve MSC survival and alter their metabolic and paracrine properties, and preconditioned MSC-derived EVs have shown enhanced cartilage-protective effects in OA models (161, 162). More recent work has combined hypoxia-mimicking preconditioning with injectable biomaterials and demonstrated enhanced MSC chondrogenic and immunomodulatory activity together with improved cartilage repair in OA models (163).Modulation of longevity- and autophagy-associated pathways such as SIRT1 may represent an additional strategy for improving MSC fitness, although evidence for genetically engineered SIRT1/FOXO1 MSCs in OA remains limited.

##### 4.4.5.2 Engineered extracellular vesicles

Cell-free EV-based approaches may provide a way to reproduce selected paracrine functions of MSC therapy while avoiding some limitations of live-cell transplantation. Chondrocyte-targeted engineered exosomes carrying miR-140 have been developed for OA and shown to deliver therapeutic cargo directly to chondrocytes (164). More recently, miR-140-engineered stem-cell-derived exosomes have been reported to reduce MMP13-associated matrix degradation, apoptosis, and inflammation while promoting cartilage repair in OA models (165).

However, EV effects are highly dependent on cellular source, cargo composition, and targeting strategy; therefore, specific miRNAs should not be assumed to exert uniform anti-senescent effects across recipient cell types.

##### 4.4.5.3 Macrophage engineering

Engineering macrophages to maintain durable pro-resolving and efferocytic functions represents a conceptually attractive strategy for preventing re-establishment of a pathological inflammatory niche (166). MerTK is a key mediator of macrophage efferocytosis, and experimental studies in other inflammatory settings demonstrate that preserving or enhancing MerTK signaling can improve efferocytosis and reduce inflammatory responses (167). However, direct evidence for genetically engineered MerTK-overexpressing macrophages as an OA therapy remains limited.

#### 4.4.6 Summary: an integrated microenvironmental reprogramming framework

In summary, accumulating evidence increasingly supports viewing OA as a dynamic pathological ecosystem wherein inflammation, cellular senescence, metabolic dysregulation, matrix degradation, uncoupled bone remodeling, and repair failure are intricately linked. Within this integrated framework, future disease-modifying interventions should not be restricted to single cytokines or individual cell types. Instead, therapeutic strategies should combinatorially modulate senescent cell burden, SASP signaling, immunometabolic fitness, inflammation resolution, and regenerative niches tailored to disease stage and predominant clinical endotypes.

This paradigm does not imply that every patient requires all therapeutic modalities simultaneously, but rather emphasizes selecting synergistic therapeutic modules matched to specific microenvironmental contexts. Recent proof-of-concept studies, such as the pCQ/SOD nanosystem targeting synovial senescent macrophages, illustrate that concurrently mitigating mitochondrial oxidative stress, suppressing the SASP, and restoring MerTK-dependent efferocytosis can confer structural protection across diverse OA models. Therefore, future DMOAD development must transition from generic anti-inflammatory agents toward spatiotemporally controlled, cell-specific, and stage-tailored microenvironmental reprogramming strategies.

## 5 Future perspectives and translational challenges

Translating osteoimmune microenvironment reprogramming from preclinical concepts into clinically effective therapies requires resolving several methodological, computational, and translational bottlenecks. While current investigations have delineated specific senescence markers and individual inflammatory pathways, whole-joint degeneration operates as an interconnected, spatially structured network. Advancing the field beyond conventional descriptive paradigms will depend on developing spatial mapping tools, computational modeling, testable resolution frameworks, and endotype-driven clinical translation strategies.

### 5.1 Spatial and computational osteoimmunology: mapping niches and deciphering intercellular dialogues

A fundamental limitation of bulk profiling and conventional single-cell RNA sequencing (scRNA-seq) is the loss of anatomical context. Tissue dissociation disrupts physical cell–cell relationships and removes information about local tissue architecture, cellular neighborhoods, and spatially constrained molecular gradients. Spatial transcriptomics (ST) has therefore become an important approach for studying OA as a spatially compartmentalized disease, with applications extending across cartilage, synovium, subchondral bone, and periarticular tissues (168). Two fundamental questions remain incompletely resolved: Where does immunosenescence occur within the intact joint, and who talks to whom across anatomical boundaries?

#### 5.1.1 Spatially resolved profiling of immunosenescent niches

Complementary spatial proteomic and multiplex imaging platforms, including co-detection by indexing (CODEX), imaging mass cytometry (IMC), and multiplex immunofluorescence, can characterize highly multiplexed protein and transcription-factor profiles while preserving tissue architecture (169). Importantly, spatial proximity or co-expression alone does not establish functional communication; spatially resolved molecular measurements should therefore be integrated with protein-level validation and functional perturbation.

#### 5.1.2 Several anatomically defined niches are particularly relevant for future investigation

##### 5.1.2.1 The cartilage–synovium interface

Determining whether senescence-associated CD4^+^CD28^-^ T cells, inflammatory macrophages, and senescence-associated stromal populations are spatially enriched at the cartilage–synovium interface and whether their proximity is associated with localized inflammatory or senescence-associated molecular signatures in adjacent chondrocytes.

##### 5.1.2.2 The osteochondral tidemark and subchondral marrow

Determining whether osteoclast-lineage cells, macrophages, vascular endothelial cells, and senescence-associated stromal populations form reproducible spatial niches around BMLs, microdamage, and regions of abnormal bone remodeling. Such studies will require particular attention to the technical challenges of spatial profiling in mineralized tissues, where decalcification, low cellularity, dense extracellular matrix, and RNA degradation can compromise spatial resolution and molecular integrity (170, 171).

##### 5.1.2.3 The infrapatellar fat pad interstitium

Determining whether macrophage-rich crown-like structures, inflammatory fibroblast populations, and local lipid/metabolic abnormalities form reproducible spatial neighborhoods within OA IPFP. Recent multiomic analysis of human IPFP has already combined single-nucleus RNA sequencing, spatial transcriptomics, and metabolomics to infer putative cell–cell communication networks, providing a practical foundation for this type of investigation (172).

Importantly, proof-of-concept studies are beginning to demonstrate the feasibility of integrated spatial multi-omics in human OA. Recent spatial proteomic and metabolomic imaging of OA synovium identified distinct spatially organized inflammatory states, illustrating how spatial molecular profiling can move beyond cell-type cataloguing toward tissue-level endotyping.

#### 5.1.3 Computational interactomics and AI-assisted communication modeling

Computational frameworks such as CellChat and NicheNet provide complementary approaches: CellChat infers putative ligand–receptor communication networks and compares their relative communication probabilities, whereas NicheNet links candidate extracellular ligands to downstream transcriptional responses in recipient cells (173, 174).

Machine-learning approaches may further help integrate high-dimensional spatial and molecular data and identify recurrent signaling motifs across disease stages or patient subgroups. Recent benchmarking and methodological studies have highlighted substantial variation in the performance and assumptions of existing cell–cell communication tools (175, 176).

#### 5.1.4 Bridging in silico predictions to causal spatial validation

A major challenge is converting computationally inferred communication into experimentally demonstrated mechanisms. CellChat and NicheNet outputs should be treated as hypothesis-generating: expression-based scores do not establish ligand secretion, receptor engagement, downstream activation, or a causal effect on the recipient cell (176, 177). Evidence for an individual ligand–receptor axis is best interpreted in tiers: (i) computationally inferred from transcriptomic data; (ii) supported by spatial co-localization and protein-level detection in anatomically plausible sender–receiver populations; and (iii) functionally validated by perturbing the ligand/receptor—for example, through ligand neutralization, receptor inhibition, or cell-specific genetic manipulation—and demonstrating a change in the recipient-cell phenotype, ideally with rescue. Axes supported only by the first tier should be described as putative, and mechanisms demonstrated only in aging or another disease context should remain extrapolations until tested in OA. Spatial CCC methods are increasingly being developed to incorporate these constraints and reduce false-positive predictions (178).

A practical validation pipeline should combine complementary steps. First, spatial imaging should confirm anatomically plausible proximity of candidate sender and receiver cells. Second, protein-level assays should verify the candidate ligand and receptor rather than relying on transcript abundance alone. Third, ligand neutralization, receptor antagonism, or cell-specific loss-of-function should test whether perturbation alters a predefined recipient-cell phenotype. Finally, rescue experiments and, where feasible, multicellular ex vivo or joint-on-chip systems can test whether the interaction persists in a more physiologically relevant setting (179). Human joint-on-chip models incorporating chondrocytes, fibroblasts, osteoblasts, and macrophages demonstrate the feasibility of testing OA-like tissue interactions in a controlled setting (180).

Ultimately, integrating spatial multi-omics with computational interactomics and functional perturbation could transform osteoimmunology from descriptive cell-state cataloguing into a spatially anchored and experimentally testable model of cellular communication.

### 5.2 The resolution failure hypothesis: why removing senescent cells alone may be insufficient

The preceding sections suggest that resolution failure may represent a systems-level consequence linking immune-state remodeling with persistent tissue damage. This convergence raises a major translational question: whether reducing the burden of senescent cells is sufficient to restore long-term tissue homeostasis. Preclinical senolytic studies have reported structural protection in some models, whereas others show pain relief without consistent structural modification. These effects are not uniform across agents or disease models (15, 18). The Resolution Failure Hypothesis integrates these observations into a testable framework. However, the causal link proposed here has not been directly established: current evidence does not show that impaired resolution capacity is responsible for limited structural recovery after senolytic intervention, and variability in senolytic outcomes alone cannot demonstrate this relationship. The hypothesis does not propose impaired inflammation resolution, defective efferocytosis, or altered SPM signaling as newly discovered biological phenomena; rather, it asks whether the persistence of these established but dysregulated processes after senescent-cell clearance may help explain why senolysis does not consistently restore whole-joint homeostasis. Under this model, senolysis may remove an important source of pathological signals without necessarily restoring the capacity of the remaining immune and stromal compartments to terminate inflammation, clear cellular debris, and re-establish tissue homeostasis.

The evidence hierarchy for the major mechanistic components of this hypothesis is summarized in **Table 5**.

**Table 5:** Evidence hierarchy supporting the resolution failure hypothesis in OA.

Component	Evidence in OA	Evidence context	Interpretation
Impaired efferocytosis	Direct human OA observation (145)	Human knee OA synovium (patient samples)	Impaired clearance is directly observed in human OA; causality for structural progression remains unresolved.
MerTK/Axl dysfunction	OA experimental context + non-OA mechanistic evidence (6, 151, 181)	OA models of senescence-associated macrophage remodeling; aging/non-OA macrophage efferocytosis studies	OA macrophage-remodeling experiments and non-OA MerTK-related mechanisms provide convergent rationale, but direct OA-specific causality of MerTK/Axl alterations is unresolved.
Resolution-related lipid mediator remodeling	Direct human OA observation (182, 183)	Human OA synovial-fluid lipidomic studies (including OA–RA comparisons)	Human OA lipidomics supports the presence and alteration of resolution-related pathways, not a universal SPM deficiency or a proven causal role in OA progression.
RvD1-mediated effects in OA models	Experimental OA evidence (147, 148)	Cultured human OA chondrocytes; experimental murine OA	OA cellular and murine experiments support anti-inflammatory and macrophage-modulating effects; restoration of overall resolution capacity or structural benefit in human OA is unproven.
RvD1 → senescence	Non-OA mechanistic evidence (149, 150, 152)	Aging and non-OA disease models	Potential senescence-modulating activity; not directly established in human OA.
Senolysis → restoration of resolution (proposed)	Hypothetical/integrative (separate OA experimental support) (6, 128)	Separate OA experimental studies of senescence-associated macrophage remodeling and efferocytosis-oriented treatment; no direct sequential test	The component interventions have OA experimental support, but it remains untested whether senolysis restores resolution capacity or whether combining both interventions improves structural repair.
Resolution failure → OA progression	Hypothetical/integrative	Cross-model synthesis; not directly tested	Proposed causal bridge; its direction, necessity and therapeutic implications require direct validation.

Evidence categories indicate the biological setting and type of support for each specified component, not proof of the entire proposed causal sequence. “Direct human OA observation” denotes associations or measurements in patient-derived OA tissue or synovial fluid; these observations do not, by themselves, establish causality. “Experimental OA evidence” refers to functional interventions in OA animal models or studies using OA-derived cells/tissues. “Non-OA mechanistic evidence” refers to mechanistic findings from aging, other skeletal disorders, or related diseases that are extrapolated to OA. “Hypothetical/integrative” denotes a causal connection proposed here but not directly tested, even when the individual components are supported by separate OA experiments. Mixed-category entries identify distinct contexts rather than implying that OA-specific causality has been demonstrated.

#### 5.2.1 Mechanistic pillars of the resolution failure hypothesis

##### 5.2.1.1 Efferocytosis failure and secondary necrosis

Inflammation resolution requires active clearance of apoptotic cells through efferocytosis. Human OA studies provide direct evidence that this process is impaired in the diseased joint: late-stage knee OA synovium contains more apoptotic cells, while synovial macrophages show both a lower proportion of cells engaging in efferocytosis and reduced uptake per macrophage. Exposure of blood-derived macrophages to OA synovial fluid can reproduce this defect, suggesting that the altered joint milieu itself can impair efferocytic function (145).

Mechanistically, aging and inflammatory signaling can reduce the activity of efferocytic receptors such as MerTK and Axl. ADAM17-mediated proteolytic cleavage is one established mechanism of MerTK inactivation, and experimental studies in aged macrophages have shown that increased ADAM17 activity promotes MerTK shedding and defective efferocytosis (181). More directly relevant to OA, a recent senotherapeutic nanoparticle study demonstrated that pCQ/SOD treatment enhanced efferocytosis of senescent synovial macrophages through a STAT3/ADAM17/MerTK axis and simultaneously reduced mitochondrial damage and SASP production, improving both aging-associated and trauma-induced OA in mice (6).

Failure to efficiently clear apoptotic cells and cellular debris may allow secondary necrosis and prolonged exposure to intracellular danger signals. Whether defective efferocytosis directly promotes NET retention and DAMP accumulation in human OA, however, remains to be established.

##### 5.2.1.2 Dysregulated specialized pro-resolving mediator signaling

Inflammation resolution is an active biochemical process regulated by SPMs. Human OA synovial-fluid studies demonstrate that both inflammatory and pro-resolving lipid pathways are active within the joint. Targeted lipidomics has identified PGE2, thromboxane-derived mediators, 17-HDHA, 18-HEPE, and other pathway markers in OA synovial fluid, together with detectable pro-resolving mediators such as RvD2. Altered 5-LOX and 15-LOX pathway activity has also been observed in OA (182).

Importantly, current evidence does not support a simple model in which OA is universally characterized by an absolute deficiency of SPMs. In a comparison of severe RA and OA, several pro-resolving lipid mediators were actually higher in RA synovial fluid than in OA, while earlier studies found evidence of active resolution pathways in OA itself (183). A more plausible model is therefore one of dysregulated resolution signaling, in which the timing, local abundance, biosynthetic flux, receptor responsiveness, and balance between inflammatory and pro-resolving pathways may be insufficient relative to the magnitude and persistence of tissue injury.

This distinction is therapeutically relevant. RvD1 has been shown to suppress inflammatory and catabolic signaling in human OA chondrocytes, including NF-κB, MAPK, MMP-13, COX-2, and oxidative-stress responses (147). Moreover, emerging clinical data from the 2026 GAUDI randomized trial indicate that SPM-enriched supplementation may influence inflammatory biomarkers and improve patient-reported outcomes, although the biomarker effects were modest and did not establish structural disease modification (184).

##### 5.2.1.3 Persistent SASP imprinting and epigenetic memory

Cellular senescence is accompanied by durable chromatin remodeling, including formation of H3K27ac-enriched enhancer and super-enhancer landscapes that support sustained SASP transcription. BRD4 recruitment to newly activated senescence-associated enhancers contributes to the expression of inflammatory SASP genes, while additional chromatin regulators can maintain the senescence-associated transcriptional state (185). Recent work further indicates that mitochondrial metabolism can directly influence H3K27ac at SASP loci, strengthening the mechanistic connection between metabolic dysfunction and persistent inflammatory transcription (186).

Whether comparable forms of durable epigenetic “memory” are established in non-senescent synovial fibroblasts, osteoclast-lineage cells, or stromal progenitors after exposure to an immunosenescent OA microenvironment remains unknown. This possibility is therefore best regarded as a testable extension of established senescence biology rather than a demonstrated mechanism in human OA.

#### 5.2.2 Major uncertainties and knowledge gaps

##### 5.2.2.1 Causality versus consequence

It remains unresolved whether impaired efferocytosis and altered SPM signaling are primary contributors to the establishment of the immunosenescent OA microenvironment or secondary consequences of chronic mechanical loading, tissue injury, and structural degeneration.

##### 5.2.2.2 Autonomous recovery after senolysis

An important unanswered question is whether selective senescent-cell clearance is sufficient to restore efferocytic capacity and resolution signaling in the remaining immune and stromal cell populations, or whether additional interventions are required to re-establish these functions.

##### 5.2.2.3 Spatiotemporal and compartmental dynamics

The spatial distribution and temporal regulation of SPM receptors—including FPR2/ALX, ChemR23, and GPR18—as well as efferocytic receptors such as MerTK and Axl, remain incompletely characterized across the synovium, cartilage, IPFP, and subchondral bone during OA progression.

Addressing these questions through stage-specific loss-of-function models, spatial lipidomics, longitudinal efferocytosis assays, and functional combination studies will help determine whether restoration of endogenous resolution capacity represents a missing therapeutic dimension beyond senescent-cell clearance alone.

##### 5.2.2.4 Experimental approaches to test the causal model

A direct test would use a factorial design in a well-characterized OA model with four groups: vehicle, senolytic monotherapy, a resolution-restoring intervention alone, and the combination. The second intervention could target macrophage efferocytosis or pro-resolving signaling (for example, an RvD1/SPM-based strategy), with its activity and exposure first confirmed in pilot experiments. Early readouts should include senescent-cell burden and SASP, macrophage efferocytosis, MerTK/Axl function, and local SPM profiles; later outcomes should include blinded cartilage histopathology, synovitis, subchondral bone microarchitecture, and pain or joint function. The hypothesis predicts that combination treatment will improve resolution-related readouts and structural outcomes beyond senolysis alone despite comparable senescent-cell clearance. Causal dependence could be probed by blocking the resolution-restoring pathway: loss of the added structural benefit under pathway blockade would support the proposed link, whereas no incremental benefit—or persistence of benefit despite blockade—would weaken it. Replication in age-associated and post-traumatic OA models would help distinguish aging-related effects from injury-driven inflammation.

### 5.3 Precision regenerative osteoimmunology: matching microenvironmental endotypes to targeted reprogramming

The limited success of DMOAD development may partly reflect the biological and clinical heterogeneity of OA. Rather than representing a single disease entity, OA encompasses multiple interacting pathological dimensions involving inflammation, tissue turnover, structural damage, metabolic dysfunction, and subchondral bone remodeling. Biomarker- and imaging-based studies have therefore begun to identify molecular and clinical endotypes that may be relevant for patient stratification and therapeutic development (187, 188). Recent exploratory work has further demonstrated the feasibility of predicting molecular endotypes from blood biomarkers and examining differential treatment effects across previously conducted OA trials (189).

However, the endotypes proposed below should not be regarded as validated clinical categories. Rather, they represent hypothesis-generating osteoimmune therapeutic archetypes derived from the mechanistic framework developed in this review. Their biological stability, overlap, prognostic value, and—most importantly—their ability to predict treatment response require prospective validation.

#### 5.3.1 Multidimensional diagnostic stratification

Precision osteoimmunology will likely require integration of molecular biomarkers, synovial-fluid analyses, quantitative imaging, and clinical phenotyping rather than reliance on a single marker. Synovial-fluid profiling is particularly attractive because it is more proximal to the joint than circulating biomarkers and may better capture local inflammatory and tissue-remodeling processes (190).

#### 5.3.2 Circulating and synovial-fluid biomarkers

Candidate biomarkers may include inflammatory and tissue-remodeling mediators such as macrophage signals, and neutrophil elastase, which have been associated with imaging-defined synovitis and OA severity (191).

#### 5.3.3 Tissue-anchored imaging readouts

HR-pQCT enables quantitative assessment of periarticular and subchondral bone microarchitecture and may provide an additional structural layer for bone-remodeling endotyping (192). 18F-NaF PET/CT or PET-MRI can provide an investigational measure of regional bone turnover and may help identify metabolically active subchondral compartments (193). The major challenge will be integrating these imaging measures with molecular data into reproducible patient-level classifiers.

#### 5.3.4 Proposed osteoimmune therapeutic archetypes

##### 5.3.4.1 Synovial inflammation-dominant endotype

###### 5.3.4.1.1 Candidate features

This proposed phenotype would be characterized by prominent MRI-detected synovitis or effusion together with local inflammatory and myeloid signatures. Candidate molecular features include macrophage-associated markers, neutrophil elastase and elevated inflammatory cytokine activity. Human studies already provide evidence for an inflammatory OA subset associated with synovial inflammation and macrophage/neutrophil-related biomarkers (194).

###### 5.3.4.1.2 Potential reprogramming strategies

Candidate interventions could include pathway-directed senomorphic approaches, resolution-oriented therapies, targeted anti-inflammatory delivery, and, where excessive NET formation is demonstrated, experimental NET-targeting strategies. Responsive hydrogels or other local delivery systems may help concentrate these interventions within inflamed synovial compartments. Such treatment matching remains hypothetical and would require prospective biomarker-defined trials.

##### 5.3.4.2 Subchondral bone remodeling-dominant endotype

###### 5.3.4.2.1 Candidate features

Mechanistically, candidate features may include altered RANKL/OPG signaling, increased osteoclast activity, abnormal vascular remodeling including type-H vessels, and osteochondral sensory nerve ingrowth. HR-pQCT studies have demonstrated measurable changes in subchondral trabecular architecture in human OA and relationships with disease stage and mechanical alignment (192).

###### 5.3.4.2.2 Potential reprogramming strategies

This phenotype could motivate investigation of bone-targeted delivery systems, osteoclast-modulating approaches, and interventions directed at osteochondral neurovascular signaling. Candidate pathways include NGF/TrkA and osteoclast-derived Netrin-1/DCC signaling, although these remain primarily preclinical targets. Structural biomaterials may additionally be considered when substantial osteochondral defects or altered load distribution are present.

##### 5.3.4.3 Cartilage senescence-dominant endotype

###### 5.3.4.3.1 Candidate features

A proposed cartilage-senescence phenotype would be characterized by a high local burden of senescence-associated chondrocyte features relative to inflammatory or bone-remodeling signatures. Candidate tissue markers could include coordinated evidence of p16^INK4a^/p21^CIP1^ expression, persistent DNA-damage signaling, altered proliferative capacity, SASP-associated mediators, and increased matrix-catabolic activity such as MMP-13 and ADAMTS-5. Importantly, no single senescence marker should be used to define this phenotype.

###### 5.3.4.3.2 Potential reprogramming strategies

This phenotype could be particularly suitable for local senolytic or senomorphic strategies combined with anabolic or regenerative interventions. D+Q and navitoclax have shown preclinical activity against senescent OA chondrocytes, while engineered EVs carrying miR-140 have been developed for chondrocyte-targeted delivery and cartilage repair (164). Whether such strategies provide greater benefit in biomarker-defined senescence-dominant patients remains unknown.

##### 5.3.4.4 Resolution- and regeneration-deficient endotype

###### 5.3.4.4.1 Candidate features

A proposed resolution-deficient phenotype would be characterized by impaired macrophage efferocytosis, altered efferocytic receptor signaling, dysregulated pro-resolving lipid mediator activity, and impaired regenerative-stromal function. Human OA studies demonstrate reduced synovial macrophage efferocytosis, providing direct evidence for one component of this phenotype (145). These features should currently be regarded as candidate biomarkers rather than validated diagnostic criteria.

###### 5.3.4.4.2 Potential reprogramming strategies

Potential interventions could combine restoration of efferocytosis, pro-resolving lipid signaling, and regenerative-niche support. Recent OA studies provide proof-of-concept for both RvD1-based macrophage modulation and nanoparticle-mediated restoration of senescent macrophage efferocytosis (6). Whether combination treatment improves structural outcomes beyond single-pathway intervention remains to be determined.

#### 5.3.5 From generic palliative care to precision DMOAD development

The application of osteoimmune endotyping to OA drug development could shift clinical trials from broad, unselected recruitment toward biologically enriched populations in which the target pathway is demonstrably active. This concept is supported by existing endotype studies and, more recently, by exploratory analyses showing that biomarker-predicted molecular endotypes can be derived and applied retrospectively to participants in prior phase II/III OA trials (189).

However, precision DMOAD development should not assume that OA can be divided into four mutually exclusive categories. The emerging literature is itself heterogeneous: some studies identify discrete biomarker-based endotypes, whereas a recent large-scale analysis of 1, 361 patients found a predominantly continuous molecular landscape rather than clearly separated clusters (195). The more realistic future model may therefore involve multidimensional endotyping, in which patients occupy overlapping positions along inflammatory, senescence-associated, bone-remodeling, metabolic, and regenerative axes.

Under this framework, composite biomarkers and advanced imaging could identify the dominant biological drivers in an individual patient and guide selection of senotherapeutic, immunometabolic, pro-resolving, bone-targeted, or regenerative interventions. The ultimate goal is not to create rigid disease categories, but to determine which pathological circuit is dominant, which therapeutic node is actionable, and when intervention is most likely to modify disease trajectory.

### 5.4 From experimental proof-of-concept to clinical translation: overcoming translational hurdles

While the therapeutic strategies outlined in Section 4 provide compelling proof-of-concept for microenvironmental reprogramming, much of the current evidence remains derived from surgically induced rodent models, *in vitro* monocultures, and engineered cellular or biomaterial systems. Translating these findings into clinically effective therapies will require careful attention to model validity, cellular heterogeneity, joint pharmacokinetics, disease stage, biomarker qualification, safety, and manufacturing.

#### 5.4.1 Preclinical model disconnect and human pathological complexity

A substantial proportion of preclinical OA research relies on surgically induced rodent models, particularly destabilization of the medial meniscus (DMM) and anterior cruciate ligament transection (ACLT). These models provide strong experimental control and are valuable for studying specific mechanisms of joint instability and tissue degeneration, but no individual OA model fully reproduces the heterogeneous, slowly progressive, whole-joint trajectory of human disease (196, 197).

Human OA develops over years to decades, an intervention that suppresses accelerated cartilage degeneration in a relatively young, otherwise healthy animal may not reproduce the therapeutic response of an older patient with established whole-joint disease (198). Future studies should therefore adopt model–target matching, selecting post-traumatic, age-related/spontaneous, metabolic, and large-animal models according to the clinical phenotype and biological mechanism being investigated.

#### 5.4.2 Senescent-cell heterogeneity and target selectivity

Cellular senescence is not a uniform biological state. Recent studies demonstrate substantial heterogeneity in senescent-cell phenotypes according to cellular origin, tissue, inducing stress, and molecular trajectory, with p16^INK4a^ and p21^CIP1^ associated populations frequently exhibiting distinct characteristics (199). Moreover, senescent cells can have context-dependent physiological functions during tissue remodeling and repair. Experimental studies have therefore raised concerns that indiscriminate senolysis may interfere with beneficial injury responses, suggesting the existence of a therapeutic window determined by dose, timing, and cellular target (200).

Therapeutic selectivity should therefore be based on cellular identity, anatomical location, persistence, survival dependency, SASP composition, and functional phenotype, rather than reliance on a single senescence marker.

#### 5.4.3 Intra-articular pharmacokinetics and delivery barriers

Therapeutic agents can undergo relatively rapid clearance through the synovial membrane and lymphatic system, while distribution into cartilage is limited by tissue structure, matrix composition, and molecular size (201, 202). Long-acting, tissue-penetrating, and microenvironment-responsive delivery systems may therefore be required to maintain therapeutically relevant local exposure while limiting systemic toxicity (203).

#### 5.4.4 Disease-stage dependence and diminishing regenerative opportunity

OA should be viewed as a continuum of declining regenerative opportunity, in which the relative contribution of inflammation, senescence, mechanical dysfunction, bone remodeling, and tissue loss changes over time. This concept argues for earlier biological stratification and intervention rather than waiting for radiographically advanced disease. Current DMOAD reviews similarly emphasize disease stage, patient selection, and sensitive outcome measures as major determinants of clinical translation (204, 205).

#### 5.4.5 Biomarker qualification, clinical endpoints, safety, and biomanufacturing

##### 5.4.5.1 Biomarker qualification

No single biomarker adequately captures the multidimensional state of an OA joint. Conventional measures such as circulating CRP and radiographic joint-space width provide useful but limited information about local inflammation, cartilage biology, subchondral remodeling, and osteoimmune activity. MRI can provide additional information on synovitis, cartilage composition, and BMLs and may therefore have an increasingly important role in DMOAD trials (206).

Future precision trials may require composite biomarker panels incorporating synovial-fluid inflammatory and tissue-remodeling markers, senescence-associated signals, lipid mediator profiles, and quantitative imaging. However, candidate biomarkers—including SPM-related lipid ratios and soluble MerTK—should currently be considered exploratory until their analytical validity, reproducibility, prognostic value, and predictive treatment-response value are prospectively qualified.

##### 5.4.5.2 Clinical endpoint alignment

A change in cartilage thickness or radiographic joint space should not be assumed to represent meaningful clinical benefit, and successful DMOAD development will likely require integrated assessment of pain, physical function, structural progression, and biological target engagement (207).

##### 5.4.5.3 Long-term safety

Chronic or repeated administration of senotherapeutic and immune-modulating interventions requires careful surveillance for delayed tissue repair, infection, altered immune surveillance, effects on hematopoietic niches, and unintended elimination or suppression of physiologically important senescent-cell populations. The context-dependent functions of senescent cells make dosing interval, treatment duration, and tissue selectivity particularly important safety parameters (200).

##### 5.4.5.4 Scalability and regulatory compliance

Cell therapies, engineered EVs, and responsive nanomaterials introduce substantial manufacturing and regulatory challenges. For MSC-derived EVs, critical issues include identity, purity, reproducibility, potency, sterility, storage stability, cargo characterization, and scalable GMP-compatible production (208). Regulatory frameworks for EV-based medicinal products also remain heterogeneous across jurisdictions, increasing the importance of well-defined critical quality attributes, comparability assays, pharmacokinetic characterization, and standardized potency testing (209, 210).

Addressing these translational challenges will be essential for moving osteoimmune reprogramming from experimental proof-of-concept toward clinically testable, stage-tailored DMOAD strategies.

## 6 Conclusion

Immunosenescence in OA should not be viewed merely as a passive accumulation of aged immune cells or an inevitable byproduct of chronological aging. Rather, it represents a multifaceted functional, metabolic, and phenotypic remodeling of both innate and adaptive immune compartments. Age-associated immune remodeling, including senescence-associated and functionally altered immune states, may contribute to persistent inflammatory signaling in OA. The strength of evidence varies substantially across immune subsets, with the most direct human OA evidence currently supporting macrophage dysfunction and impaired efferocytosis.

Crucially, an important contributor to progressive joint pathology may be the way age-associated immune remodeling alters intercellular communication across the joint. Senescence-associated and age-remodeled immune states can generate inflammatory, damage-associated, and metabolic signals that interact with cartilage, synovial, subchondral, IPFP, and progenitor compartments, establishing self-reinforcing feedback loops that may promote cartilage breakdown, chronic synovitis, abnormal bone remodeling, and impaired regeneration.

To achieve authentic disease modification, future DMOADs must move beyond single-cytokine blockade and empirical symptom suppression. Effective microenvironmental reprogramming requires multifaceted, stage-specific strategies that concurrently reduce pathogenic senescent-cell burden, restore cellular metabolic fitness, re-activate endogenous inflammation resolution through efferocytosis and SPMs, and rebuild permissive niches for tissue regeneration. By matching patient-specific osteoimmune endotypes with smart biomaterial delivery systems and cell rejuvenation platforms, precision osteoimmunology may offer new opportunities to slow structural joint deterioration and improve long-term joint function.
